# Response of soybean root exudates and related metabolic pathways to low phosphorus stress

**DOI:** 10.1371/journal.pone.0314256

**Published:** 2024-12-05

**Authors:** Yubo Yao, Hongmei Yuan, Dandan Liu, Lili Cheng

**Affiliations:** Institute of Industrial Crops, Heilongjiang Academy of Agricultural Sciences, Harbin, China; Arish university, Faculty of agricultural and environmental sciences, EGYPT

## Abstract

Phosphorus (P) is an essential elemental nutrient required in high abundance for robust soybean growth and development. Low P stress negatively impacts plant physiological and biochemical processes, such as photosynthesis, respiration, and energy transfer. Soybean roots play key roles in plant adaptive responses to P stress and other soil-related environmental stressors. Study the changes in soybean root exudates and differences in related metabolic pathways under low phosphorus stress, analyzing the response mechanism of soybean roots to phosphorus stress from the perspective of root exudates, which provide a theoretical basis for further analyzing the physiological mechanism of phosphorus stress on soybean. In this study, soybean roots were exposed to three phosphate levels: 1 mg/L (P stress), 11 mg/L (P stress) and 31 mg/L (Normal P) for 10 days and 20 days, then root exudates were analyzed via ultra-high-performance liquid chromatography-mass spectrometry to identify effects of P stress on root metabolite profiles and associated metabolic pathways. Our results revealed that with increasing P stress severity and/or duration, soybean roots produced altered types, quantities, and increased numbers of exudate metabolites (DMs in the P1 group were primarily upregulated, whereas those in the P11 group were predominately downregulated) caused by changes in regulation of activities of numerous metabolic pathways. These pathways had functions related to environmental adaptation, energy metabolism, and scavenging of reactive oxygen species and primarily included amino acid, flavonoid, and nicotinate and nicotinamide metabolic pathways and pathways related to isoquinoline alkaloid biosynthesis, sugar catabolism, and phospholipid metabolism. These metabolites and metabolic pathways lay a foundation to support further investigations of physiological mechanisms underlying the soybean root response to P deficiency.

## Introduction

Phosphorus (P), an essential nutrient for plant growth and development, plays a central role in energy metabolism, information transfer, and synthesis of nucleic acids and membranes [1–3]. Plants grown in P-deficient soil often struggle to thrive due to adverse effects of P stress on various life processes, resulting in growth retardation, weakened structural features, and premature reproductive organ development that ultimately lead to reduced crop yields [1]. Consequently, there is an urgent need for research to deepen our understanding of physiological and molecular mechanisms underlying plant adaptive responses to P stress toward achieving sustainable agricultural development [4].

Plant roots continuously adjust their physiological and structural characteristics in response to nutrient-deficient soil conditions, as the root system is typically the first plant organ affected by changes in soil nutrient content [5–7]. Given that P is crucial for plant growth, plants have evolved strategies to enhance soil P availability and accessibility that involve intensive root formation, turnover, and/or morphological changes induced by exposure to P-deficient soil [8, 9]. Under the low phosphorus stress, the soybean genotype with high phosphorus efficiency increased the total root length by increasing the length of the lateral root, which improved plant growth, phosphorus efficiency, and enhanced the ability to resist the low phosphorus stress [10]. Additionally, plants respond to shifts in soil nutrient levels by releasing root exudates containing various organic compounds that increase nutrient availability and uptake by roots [11, 12]. Therefore, monitoring of indicators related to root system growth and development and root exudate composition can provide valuable insights into nutrient acquisition mechanisms of plant.

Root exudates play pivotal roles in mediating the movement of nutrients and other substances into and out of roots, all while regulating these processes and nurturing the vitality of rhizosphere ecosystems [13]. Importantly, root exudates released by plants grown in P-deficient soil contain substances that can substantially enhance crop growth. These substances achieve this beneficial effect by increasing soil P availability and subsequent utilization by plants through their effects on rhizosphere physical, chemical, and biological properties [14]. Moreover, studies have demonstrated that compositions of plant root exudates change in response to shifts in soil nutrient composition [15]. Alfalfa root exudates enhance the availability of soil P, increase the content of Olsen-P, and have a certain activation effect on Ca_2_-P, Ca_8_-P, Al-P, and Fe-P [16].

Alternatively, some plants respond to low P stress by secreting increased amounts of sugars and amino acids into the rhizosphere to improve P absorption [17]. For example, Koeppe et al. reported that sunflower roots subjected to low P stress secreted significantly increased quantities of a phenolic substance that promotes P nutrient uptake [18]. In a separate study of mung bean and maize root exudates, Al-Deliamy and Ameer observed significantly lower concentrations of most components in exudates of plants grown in soil containing several distinct types of fertilizers as compared to concentrations in exudates of unfertilized plants [19].

Notably, roots of soybean plants grown in P-deficient soil are known to secrete significant quantities of protons and organic acids into the external environment [20]. Root exudates play crucial roles in facilitating the exchange of energy, matter, and information between plants and soil, serving as a fundamental adaptive response to environmental stress. Therefore, using metabolomics techniques to analyze the changes in the types and quantities of soybean root exudates under low P stress, as well as the differences in metabolic pathways involved, and to analyze the response of soybean to low P stress from the perspective of root exudates. Enriching the theoretical research on the impact of phosphorus stress on soybeans.

## Materials and methods

### Plant materials and sampling

This experiment was carried out in the experimental base of Heilongjiang academy of agricultural sciences, Harbin city, Heilongjiang Province, China (126°63’ E, 45°69’ N). Soybean (Suinong 14, SN14) plants were grown in sand medium in pots. The nutrient composition and concentration (mg/L) of the nutrient solution were as follows: (NH_4_)_2_SO_4_, 235.80; MgSO_4_, 240.00; CaCl_2_, 220.00; Na_2_MoO_4_·H_2_O, 0.03; CuSO_4_·5H_2_O, 0.08; ZnSO_4_·7H_2_O, 0.22; MnCl_2_·4H_2_O, 4.90; H_3_BO_3_, 2.86; FeSO_4_·7H_2_O, 5.57; and Na_2_EDTA, 7.45; P levels: P1 (P stress), KH_2_PO_4_, 4.39, K_2_SO_4_, 42.00, KCl, 36.00; P11 (P stress), KH_2_PO_4_, 48.26, K_2_SO_4_, 28.00, KCl, 24.00; P31 (normal P), KH_2_PO_4_, 136.00.

Different P levels treated 10 days and 20 days, the root exudate solution were sampled. Before the vegetative cotyledon stage (VC, unfolded cotyledons), only 500 mL distilled water was supplied to plants once per day. From VC to V_3_ (third trifoliate leafs) stage, P31 nutrient solution was supplied, fromV_3_ different P treatments were started, and 500 mL nutrient solution was supplied once a day before R_1_ (flowering stage), 500 mL nutrient solution was supplied two times a day from R_1_.

Rhizobium inoculation was performed when opposite true leaves completely opened as follows: field soybean nodules from plants grown in Harbin Heilongjiang province were collected during the previous year and stored in a refrigerator. They were ground and added to the nutrient solution, at a rate of approximately 5 g of nodules per liter. Inoculation of soybean plants performed on five consecutive days to assure that each plant well inoculated.

The root of the whole plant was taken and quickly washed with flowing deionized water to remove sand and nutrient solution. Immerse the washed root into a glass container with 250 mL deionized water. The root in water for 10 h under the same climatic conditions as the plant growth, and collected the root exudate solution (6 replicate samples for each treatment) and frozen immediately in liquid nitrogen and stored at −80°C, and then used for metabolomics analysis.

### Metabolites extraction

The samples were thawed on ice. After 30 s vortex, 4000 μL aliquot of individual samples were transferred to an eppendorf tube and freeze-dried. 200 μL of extract solution (methanol/water = 3:1, precooled at -40°C, containing internal standard) were added to the samples. After 30 s vortex, the samples were sonicated for 10 min in ice-water bath. Then the samples were centrifuged at 12000 rpm (RCF = 13800 (×g), R = 8.6 cm) for 15 min at 4°C. The supernatant was carefully filtered through a 0.22 μm microporous membrane, and take 20 μL from each sample and pooling as QC samples. Stored at -80°C until the UHPLC-MS (Ultra high performance liquid chromatog-mass spectrometry) analysis.

### UHPLC- MS analysis

The UHPLC separation was carried out using an EXIONLC System (Sciex). Perform chromatographic separation of the target compound using Waters UPLC liquid chromatography column (Water Acquity UPLC HSS T3 1.8um 2.1×100 mm). The mobile phase A was 0.1% formic acid in water, and the mobile phase B was acetonitrile. The column temperature was set at 40°C. The auto-sampler temperature was set at 4°C and the injection volume was 2 μL.

A Sciex QTrap 6500+ (Sciex Technologies), was applied for assay development. Typical ion source parameters were: IonSpray Voltage: +5500/-4500 V, Curtain Gas: 35 psi, Temperature: 400°C, Ion Source Gas 1:60 psi, Ion Source Gas 2: 60 psi, DP: ± 100 V.

### Data analysis

SCIEX analyst work station software (Version 1.6.3) was employed for MRM data acquisition and processing. MS raw data (.wiff) files were converted to the TXT format using MSconventer. In-house R program and database were applied to peak detection and annotation [21–23].

### Real-time quantitative PCR analysis

The RNA extracts of roots were used to synthesize cDNA, 20 μL reaction volume containing 1 μg of RNA template. Real-Time PCR was performed according to the instructions provided with the TB Green® Premix Ex Taq™ II kit (Tli RNase H Plus) (TaKaRa) in a 20 μL PCR reaction volum. Three technical replicates and three biological replicates were performed in the experiments. Using GmActin as the internal control. According to the Ct values, the relative expression levels were calculated using the 2^−ΔΔCt^ method.

## Results

### Differential metabolites (DMs) in root exudates under P stress

**Fig 1:**
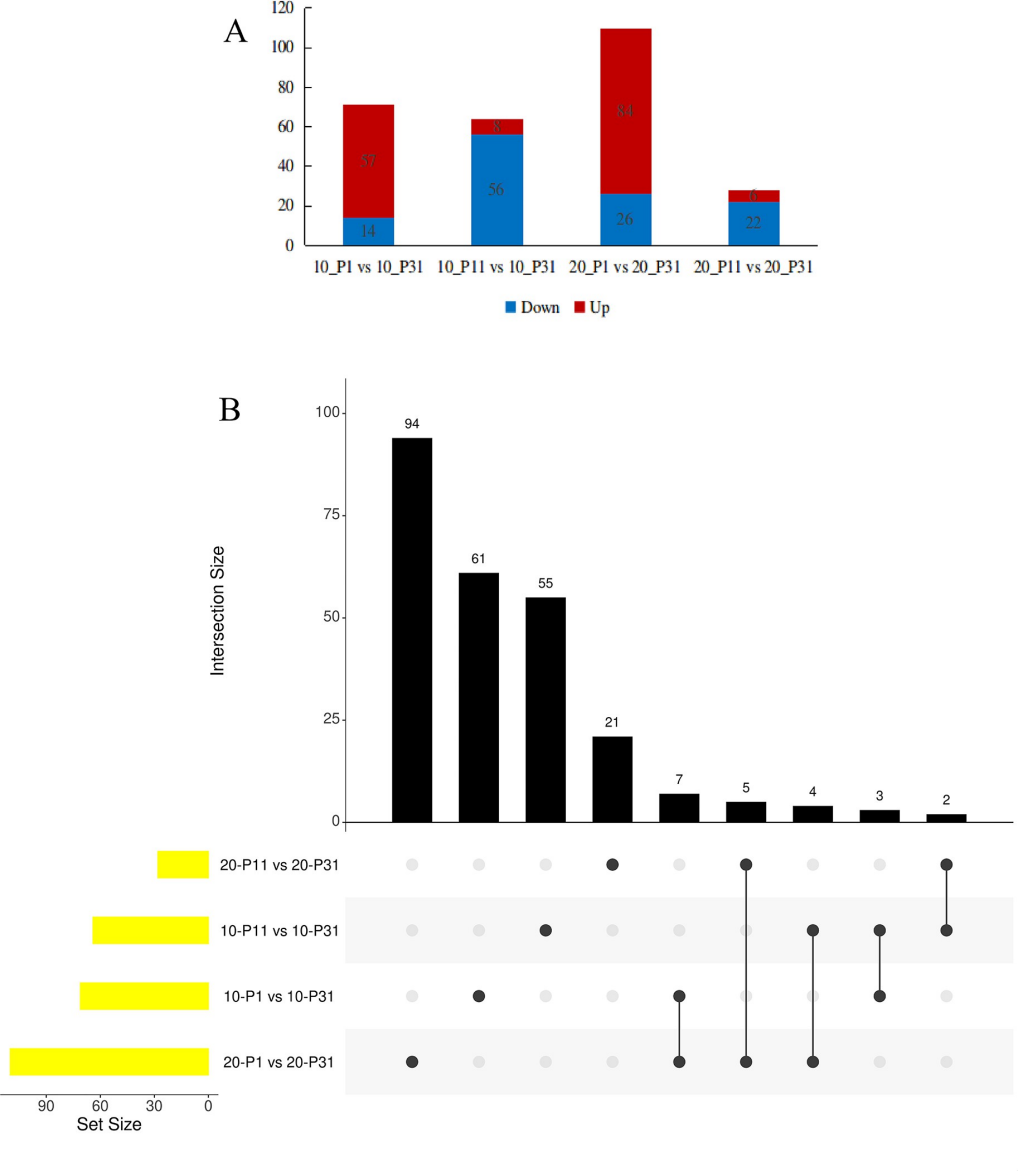
Analysis of different metabolites. (A) Number of different metabolites, up-regulated and down-regulated expression between 10_P1 vs 10_P31, 10_P11 vs 10_P31, 20_P1 vs 20_P31 and 20_P11 vs 20_P31; (B) Venn analysis of different metabolites. The yellow column represents the number of different metabolites in the comparison group, the black histogram represents the number of metabolites corresponding to the intersection difference set of Venn analysis, and the black dot represents the comparison group corresponding to the intersection difference set.

Comparisons of metabolite profiles of root exudates secreted by soybean plants under P stress vs those secreted by unstressed plants (grown in soil containing 31 mg/L P) yielded a total of 1450 metabolites that included alkaloids, flavonoids, terpenoids, phenols, amino acids and derivatives, phenylpropanoids, organooxygen compounds, steroids and steroid derivatives, and others (listed in S1 Table). Of these DMs, 71 (57 upregulated, 14 downregulated), 64 (8 upregulated, 56 downregulated), 110 (84 upregulated, 26 downregulated), and 28 (6 upregulated, 22 downregulated) were detected in pairwise comparisons 10_P1 vs 10_P31, 10_P11 vs 10_P31, 20_P1 vs 20_P31, and 20_P11 vs 20_P31, respectively, as based on screening threshold cutoffs of variable importance in projection (VIP) > 1.0 and P-value < 0.05 (Fig 1 and S2 Table).

DMs meeting criteria of VIP > 1.0, P-value < 0.05, and a fold-change >2 or <0.5 were selected for further analysis. Based on these criteria, we identified 28 DMs in the pairwise comparisons of the 10_P1 vs 10_P31 groups (21 increased, 7 decreased), 40 DMs for the 10_P11 vs 10_P31 groups (5 increased, 35 decreased), 54 DMs for the 20_P1 vs 20_P31 groups (42increased, 12 decreased), and 10 DMs for the 20_P11 vs 20_P31 groups (2 increased, 8 decreased). These DMs are potentially involved in the soybean root response to P stress and thus analyzed further.

### Analysis of DMs

Pairwise comparisons between P1 and P11 (10 days and 20 days) with P31, revealed differences in DMs chemical and regulatory features of soybean root exudates (S3 Table). For the 10_P1 vs 10_P31 comparison, 28 DMs were identified that belonged to 15 chemical categories, which mainly included flavonoids (21.43%), alkaloids (14.29%), terpenoids (10.71%), phenols (7.14%), nucleotides and their derivatives (7.14%), and lignans (7.14%). Regarding abundance of specific metabolites within each of these categories, 83.33% were increased, 75.00% were increased, 100.00% were increased, 100.00% were decreased, 100.00% were increased, and 100% were increased, respectively.

For the 10_P11 vs 10_P31 comparison, 40 DMs were identified that belonged to 18 chemical categories that mainly included terpenoids (20.00%), flavonoids (15.00%), phenols (12.50%), phenylpropanoids (7.50%), lignans (7.50%), organooxygen compounds (5.00%), and steroids and steroid derivatives (5.00%). Again, regarding abundance of metabolites within these categories, 75.00%, 100.00%, 60.00%, 100.00%, 66.67%, 100.00%, and 100.00% decreased, respectively.

For the 20_P1 vs 20_P31 comparison, 54 DMs were identified that belonged to 18 chemical categories that mainly included flavonoids (22.22%), terpenoids (16.67%), alkaloids (14.81%), phenols (7.41%), organic acids and derivatives (5.56%), and fatty acyls (5.56%). With respect to abundance of metabolites in these categories, 66.67%, 88.89%, 75.00%, 75.00%, 100.00% and 100.00% increased, respectively.

For the 20_P11 vs 20_P31 comparison, 10 DMs were identified that belonged to 7 chemical categories that mainly included alkaloids (30.00%), phenols (20.00%), benzene and substituted derivatives (10.00%), nucleotide and its derivates (10.00%), flavonoids (10.00%), terpenoids (10.00%), and organic acids and derivatives (10.00%). Regarding abundance of metabolites within these categories, 100.00% decreased, 50.00% and 100.00% increased, 100.00%, 100.00%, 100.00% and 100.00% decreased, respectively. Taken together, the results obtained from the abovementioned four comparisons revealed the identities of soybean root exudates DMs as influenced by P stress of variable severity and duration.

For each pairwise comparison group, corresponding ratios of quantitative values of corresponding DMs were calculated then DMs undergoing significant changes analyzed further. The results of this analysis were visualized as matchstick diagrams displaying the top-ranked 15 metabolites with most markedly increased or decreased abundances (relative to P31) (S1 Fig). For each top-ranked DM, values of correlation coefficients were calculated using the Pearson method and presented as a heat map (S2 Fig). For each pairwise comparison group, chemical classification and source of DMs were performed, then Chord visualization of DMs was performed using the Spearman method (S3 Fig).

### KEGG annotation and metabolic pathway analysis of DMs

37, 39, 49 and 23 KEGG pathways were associated with DMs identified from the pairwise comparisons 10_P1 vs 10_P31, 10_P11 vs 10_P31, 20_P1 vs 20_P31 and 20_P11 vs 20_P31, which included 30, 28, 56 and 10 compounds, respectively (Table 1). Total numbers of DMs obtained for each comparison, the enriched KEGG metabolic pathway ID number for each DM, and the percentage of the total annotated DMs belonging to a given metabolic pathway are presented in S4 Fig.

**Table 1:** Pairwise comparison of KEGG pathways and compound differences.

Numbers	KEEG pathways	Compounds	Up-regulated	Down-regulated
10_P1 vs 10_P31	37	30	25	5
10_P11 vs 10_P31	39	28	1	27
20_P1 vs 20_P31	49	56	41	15
20_P11 vs 20_P31	23	10	1	9

KEGG metabolic pathways associated with the highest numbers of DMs included biosynthesis of secondary metabolites (gmx01110), metabolic pathways (gmx01100), isoflavonoid biosynthesis (gmx00943), biosynthesis of various plant secondary metabolites (gmx00999), flavonoid biosynthesis (gmx00941), ABC transporters (gmx02010), 2-Oxocarboxylic acid metabolism (gmx01210), biosynthesis of amino acids (gmx01230), pyrimidine metabolism (gmx00240), tropane, and piperidine and pyridine alkaloid biosynthesis (gmx00960). The numbers of DMs differing among the four comparisons were provided (S4 Table). Root exudates respond through the above metabolic pathways, when the soybean subjected to P stress. Notably, abundances of root DMs belonging to these metabolic pathways were mainly upregulated in the P1 (10 d and 20 d) group and downregulated in the P11 (10 d and 20 d) group.

**Fig 2:**
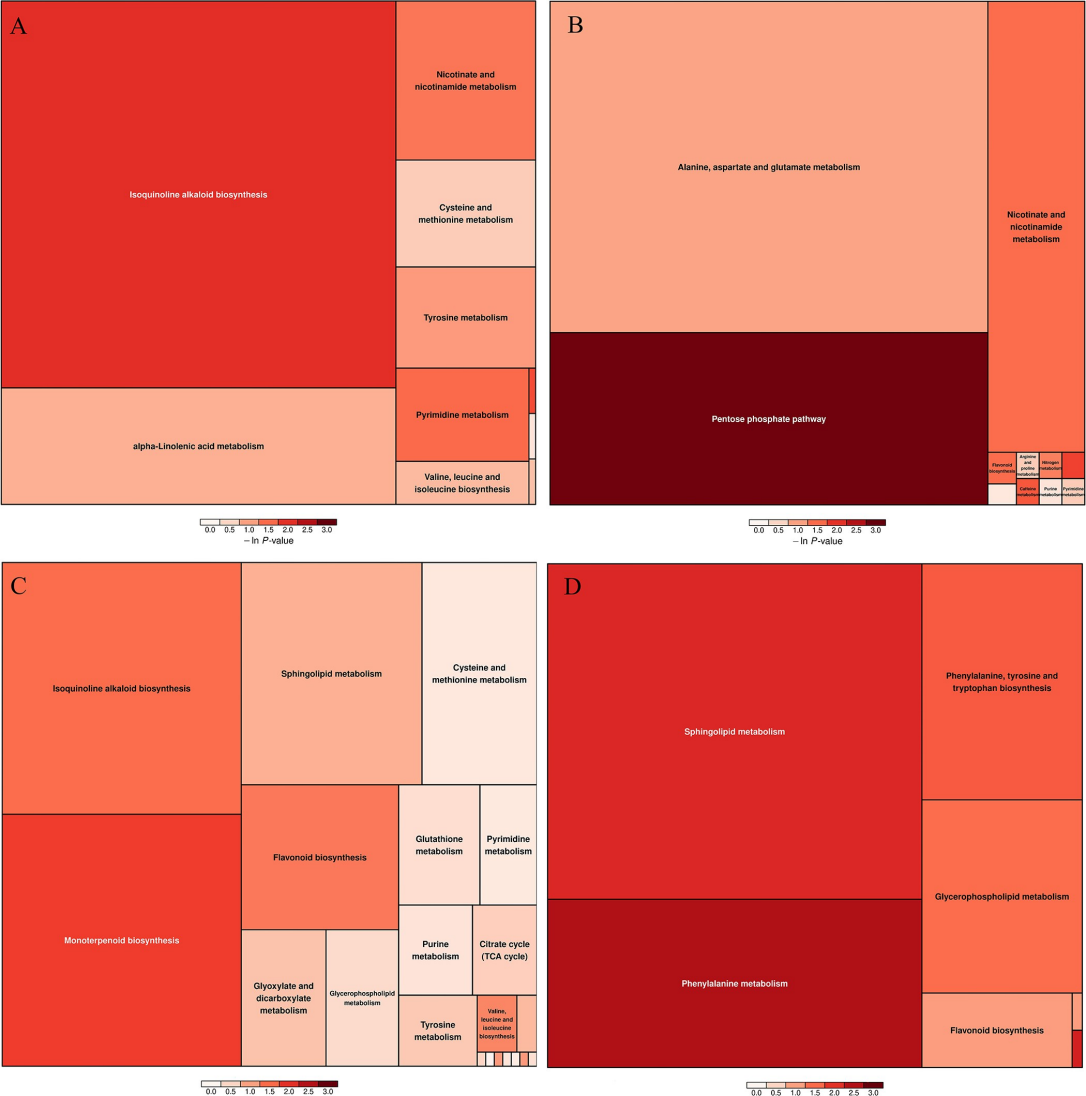
Pathway analysis results. A 10_P1 vs 10_P31; B 10_P11 vs 10_P31; C 20_P1 vs 20_P31; D 20_P11 vs 20_P31.

Results of pathway enrichment and topological analyses of these pathways revealed 10, 11, 21, and 7 key pathways associated with soybean root system responses to P stress that were identified based on DM profiles of comparisons 10_P1 vs 10_P31, 10_P11 vs 10_P31, 20_P1 vs 20_P31, and 20_P11 vs 20_P31, respectively (Fig 2). For 10_P1 vs 10_P31, key pathways included isoquinoline alkaloid biosynthesis, pyrimidine metabolism, nicotinate and nicotinamide metabolism, tyrosine metabolism, alpha-linolenic acid metabolism, valine, leucine and isoleucine biosynthesis, and cysteine and methionine metabolism. For 10_P11 vs 10_P31, key pathways included pentose phosphate pathway, nicotinate and nicotinamide metabolism, flavonoid biosynthesis, alanine, aspartate and glutamate metabolism. For 20_P1 vs 20_P31, key pathways included monoterpenoid biosynthesis, isoquinoline alkaloid biosynthesis, flavonoid biosynthesis, valine, leucine and isoleucine biosynthesis, sphingolipid metabolism, arginine and proline metabolism, glyoxylate and dicarboxylate metabolism, tyrosine metabolism, tricarboxylic acid cycle (TCA cycle), glycerophospholipid metabolism, and glutathione metabolism. For 20_P11 vs 20_P31, key pathways included phenylalanine metabolism, sphingolipid metabolism, phenylalanine, tyrosine and tryptophan biosynthes, glycerophospholipid metabolism, and flavonoid biosynthesis. Taken together, these metabolic pathways may offer valuable insights into the mechanisms by which soybean plants adapt to P stress. The pathway identified for each pairwise comparison, associated metabolite compounds and their relative abundance changes (increased or decreased) were different and shown in S5 Table.

Each rectangle in the rectangular tree represents a metabolic pathway. The size of the block represents the size of the influence factor of the pathway in topology analysis. The larger the size, the greater the influence factor; the color of the block represents the P-value of enrichment analysis (take the negative natural logarithm, namely—ln (p)). The darker the color, the smaller the P value, and the more significant the enrichment degree.

## Discussion

The term “root exudate” used to describe organic compounds released by plant roots into the surrounding medium during plant growth and development. Notably, root exudates typically sequester over 10% of photosynthesis captured carbon as organic compounds that include sugars, organic acids, fatty acids, amino acids, and others [24–27]. Consequently, root exudates play key roles in regulating rhizosphere micro ecological functions and provide the main medium for communication between plants and soil. Moreover, they play important roles in rhizosphere element-cycle, plant nutrient-absorption, and rhizosphere microbial community-shaping processes [28]. Furthermore, plants adapt to P stress and other environmental stresses by releasing root exudates containing different quantities and types of organic and inorganic compounds and other substances [29–35].

### Changes in root exudate compositions associated with soybean exposure to P stress of varying severity and duration

P plays a vital role in the normal physiological and biochemical functions of plants [36]. Consequently, when plants grown in P-deficient soil, adaptive responses are triggered to help them cope with P stress. These responses often involve root system adaptations involving root exudate compositional changes [37–44]. For example, studies of Arabidopsis and white lupine plants under P stress have revealed significant changes in root exudate levels of various phosphorylated carbohydrate compounds [42, 45]. Moreover, a study conducted by Mo demonstrated the detection of 155 DMs in soybean roots under P stress as based on comparisons with the metabolite profile of unstressed roots, including 36 lipids, 26 flavonoids, 18 amino acids and their derivatives, and 17 nucleic acids and their derivatives [46]. Meanwhile, root systems have also been shown to adapt to P stress by secreting organic acids and acid phosphatases [47, 48], with results of one such study demonstrating that the secretion of malic acid and citric acid were promoted by root systems of kidney bean, white lupine, and soybean plants under P stress [29, 33, 34]. The concentrations of amino acids and organic acids in root exudates were higher at P0, which suggested that soybean roots actively release metabolites in response to P deficiency [49]. In this study, we found that P stress severity (P1 and P11) and duration (10 and 20 days) significantly impacted the types and quantities of root exudate compounds, which were the same as Tawaraya’s opinions. The responses of soybean root exudation to P deficiency were different among growth periods and metabolites, whereby greater DM numbers were found in P1 than in P11 root exudates. Interestingly, as the duration of P stress increased, the numbers of DMs in P1 exudates showed an upward trend, while those of P11 exudates declined (Fig 1A and S2 Table). These findings collectively suggest that as the severity or duration of P stress increases, changes in soybean root exudates reflect altered activities of increasing numbers of metabolic pathways, resulting in the generation of a greater variety of distinct types of DMs as an adaptive response to P stress. And some DMs, such as alkaloids, flavonoids, amino acids and derivatives, are the same with that in nodules under P stress [50], which indicate the presence of an outward transport system of these metabolites in soybean roots [49].

We also observed an interesting trend in that DM profiles of root exudates differed depending on whether plants were exposed to P stress for either 10 or 20 days. Ultimately, we identified 3, 5, 7, and 2 identical DMs from the following pairwise comparisons: 10_P1 vs 10_P31 and 10_P11 vs 10_P31; 20_P1 vs 20_P31 and 20_P11 vs 20_P31; 10_P1 vs 10_P31 and 20_P1 vs 20_P31; and 10_P11 vs 10_P31 and 20_P11 vs 20_P31, respectively (Fig 1B). Interestingly, it was observed that DMs in the P1 group were primarily upregulated, whereas those in the P11 group were predominately downregulated. This divergence in root exudate responses suggests that soybean roots employ different mechanisms and pathways to adapt to varying degrees of P stress.

With regard to the above mentioned DMs, in 10_P1 vs 10_P31, malic acid level was positively correlated with sesartemin level; in 10_P11 vs 10_P31, beta-asarone level was positively correlated with alpha-hexylcinnamaldehyde and soyasapogenol C levels, D-ribose level was positively correlated with D-arabinose level, and actinidic acid level was negatively correlated with 2-ketobutyric acid level; in 20_P1 vs 20_P31, formononetin level was positively correlated with medicarpin and ononin levels; in 20_P11 vs 20_P31, tectorigenin level was positively correlated with 1’-acetoxychavicol acetate and myo-inositol levels, myo-inositol level was positively correlated with liriodenine level, 4’-O-methylirenolone level was positively correlated with corynanthine level, and trimethoprim level was negatively correlated with O-phosphorylethanolamine level (S3 Fig). These differential metabolites respond to phosphorus stress in a synergistic manner.

### P stress-induced changes in soybean root exudate flavonoid composition

Flavonoids, a prominent category of polyphenolic compounds, rank among the most prevalent secondary metabolites encountered in living organisms. This diverse class encompasses anthocyanins, flavanols, flavonoids, isoflavones, and flavanones, each with vital roles in plant growth, development, and plant responses to both biotic and abiotic stressors [51].

The research conducted by Mo demonstrated that exposure of soybean plants to P stress led to the accumulation of 26 distinct classes of flavonoid metabolites in roots [46]. Particularly striking was the significant divergence in response profiles of individual metabolites within the same flavonoid class in roots of plants exposed to P stress. These results indicate that P stress strongly affects activities of pathways related to soybean flavonoid metabolic processes. P limitation leads to upregulated expression of genes and accumulation of secondary metabolites phenylpropanoids, flavonoid and their glycosides, and anthocyanin [45]. In the symbiotic process between rhizo bia and legumes, flavonoids promote recognition and infection between rhizobia and roots to achieve nodulation and nitrogen fixation. Metabolomics studies demonstrated that more favonoids were involved in metabolic processes in soybean nodules under P stress [50]. Results obtained herein revealed that P stress exerted significant effects on root exudate flavonoid composition and quantity. Importantly, root exudates of plants subjected to P stress of different severities and durations exhibited differences in DM levels and numbers, as well as increasing numbers of distinct flavonoid DMs with increasing P stress severity and/or duration. The flavonoids DMs were mainly upregulation in P1, while downregulation in P11 (Table 2). 108 types of flavonoids and related metabolites were detected under P stress, including anthocyanins, flavones, flavonols, flavanones, isoflavones and derivatives of flavonoids [46]. We found that there was one flavanone (Xanthohumol) upregulation in 10_P11 vs 10_P31 and flavanoid DMs kaempferol-3-O-rutinoside and pinocembrin were downregulated in 10_P11 vs 10_P31 and 20_P1 vs 20_P31, respectively, as consistent with results reported by Mo [46].

**Table 2:** Differences in the quantity of flavonoids among comparisons.

Treatments	Numbers	Up-regulated	Down-regulated
10_P1 vs 10_P31	14	12	2
20_P1 vs 20_P31	18	12	6
10_P11 vs 10_P31	9	1	8
20_P11 vs 20_P31	3	1	2

**Fig 3:**
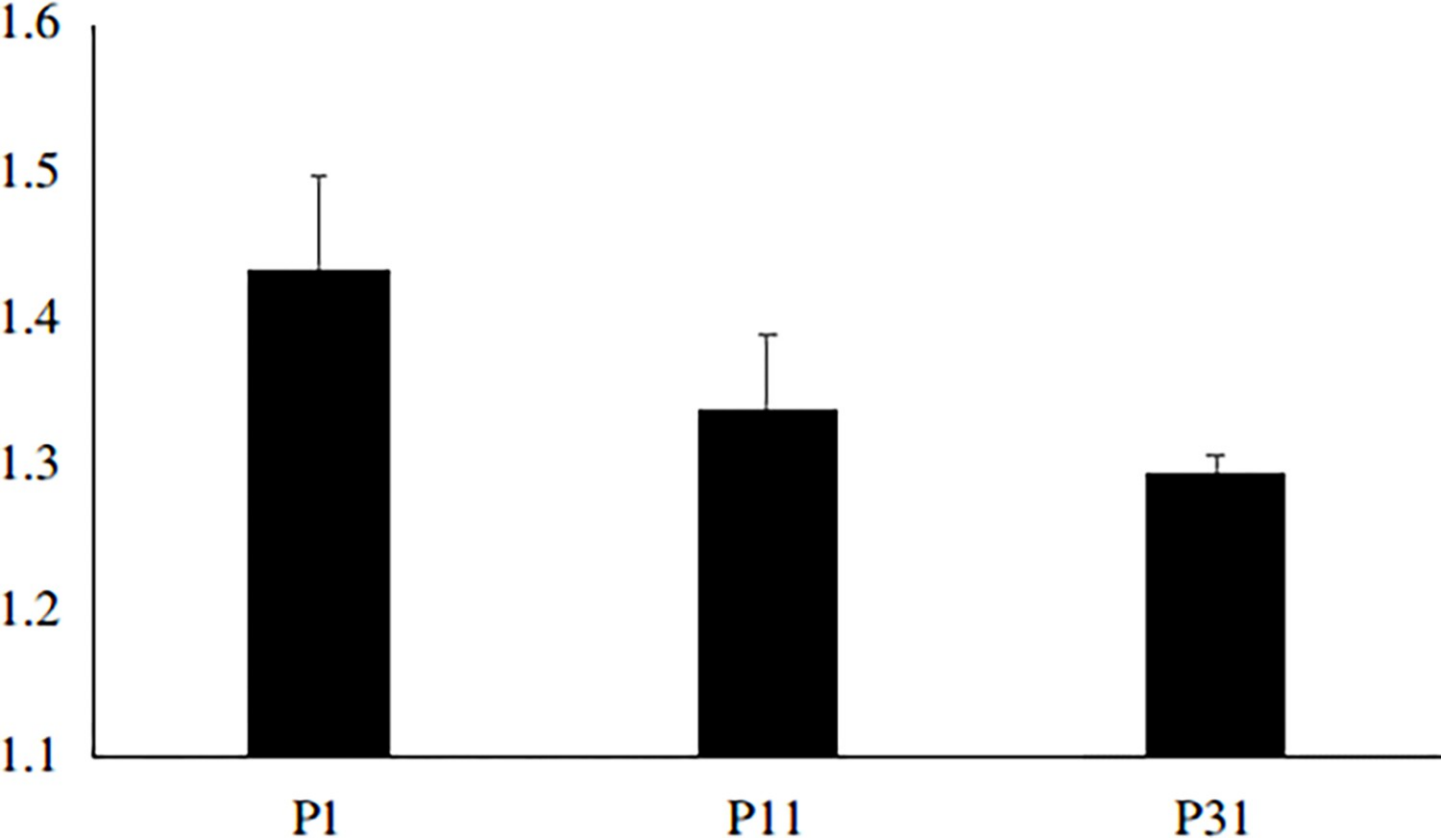
Real-time quantitative PCR analysis of GmSK2-8 in soybean root.

Roots of leguminous host crops release flavonoids into the rhizosphere environment, which can induce recognition of nodulation factors in rhizobia [52]. The fertilizer nitrogen influenced nodulation factor recognition (GmNFR1A, GmN1N1a and GmN1N2a) in the roots at V_1_ stage [53]. Cabeza et al. found that P deficiency reduced the expression of flavonoid synthesis related genes in alfalfa roots [54]. He et al. found that GmSK2-8 is strongly induced in soybean under high-salt conditions, while GmSK2-8 could interact with GmNSP1a and GmNSP1b; these key transcription factors are essential for rhizobial infection, nodule initiation, and symbiotic gene expression in soybean, providing novel targets for improving symbiotic nitrogen fixation under environmental stress conditions in soybean and possibly other legumes [55]. In order to analyze changes of GmSK2-8 under P stress, we employed real-time quantitative PCR analysis to examine RNA-level expression of the GmSK2-8-encoding gene in soybean roots following 10 days of P stress exposure. Our results revealed the relative expression level of GmSK2-8 significantly increased in P1 treatment (Fig 3). Nevertheless, further research is warranted to establish a more comprehensive understanding of the relationship between changes in flavonoid levels in soybean roots and the expression of the GmSK2-8-encoding gene under conditions of P stress.

### Metabolic pathway analysis

Analysis of DMs identified in four pairwise comparisons, 10_P1 vs 10_P31, 10_P11 vs 10_P31, 20_P1 vs 20_P31, and 20_P11 vs 20_P31, prompted us to speculate that specific soybean root response mechanisms and pathways triggered by P stress may depend on P stress severity. Building upon this premise, we further analyzed soybean root P stress-induced metabolic pathway changes. Our results indicated that P stress exerted a significant influence on the activation of various pathways, including those related to amino acid metabolism, flavonoid metabolism, nicotinate and nicotinamide metabolism, isoquinoline alkaloid biosynthesis, sugar catabolism, and phospholipid metabolism. Some of these metabolic pathways are the same as those in nodules under P stress, which indicate the presence of a transport system of metabolites between soybean roots and nodules.

Amino acids play pivotal roles as regulatory factors that control essential cellular processes, including cell signaling, gene expression, and protein phosphorylation cascades. These regulatory functions extend to a wide range of biological activities, encompassing hormone metabolism, energy metabolism, neurotransmission, cell growth, nucleotide synthesis, and nitrogen metabolism. Additionally, amino acids and their derivatives serve as precursors of molecules that participate in various plant physiological processes, including responses to drought stress, heavy metal stress, and plant disease resistance [56–58].

The results of this study underscore the role of the amino acid metabolic pathway in regulating the soybean response to P stress by highlighting its importance in the adaptation of soybean roots to low P conditions. Importantly, as the severity and duration of P stress increased, we found a corresponding increase in the production of DMs linked to a broader array of metabolic pathways (Table 3). Notably, when P1 stress treated for 20 days, the DM cysteinylglycine was upregulated and involved in the glutathione metabolism pathway. Reactive oxygen species (ROS) stress is a common cause of plant damage under abiotic stress [59], and the glutathione metabolism pathway is an important part of the antioxidant system for ROS clearance in plants, regulating the balance of intracellular ROS [60]. Therefore, when P1 stress applied for 20 days, ROS scavenging-related metabolites and metabolic pathways already activated in soybean root exudates.

**Table 3:** Amino acid metabolism pathways and differential metabolites.

Treatments	Pathway	Hits Cpd
10_P1 vs 10_P31	Cysteine and methionine metabolism Tyrosine metabolism Valine, leucine and isoleucine biosynthesis	5’-S-Methyl-5’-thioadenosine ↑ Dopamine ↓ 3-Isopropylmalate ↑
20_P1 vs 20_P31	Arginine and proline metabolism Glutathione metabolism Tyrosine metabolism Valine, leucine and isoleucine biosynthesis	N-Acetyl-L-glutamate 5-semialdehyde ↑ S-Adenosylmethionine ↑ Cysteinylglycine ↑ Dopamine ↓ 3-Isopropylmalate ↑ 2-Isopropyl-3-oxosuccinate ↑
10_P11 vs 10_P31	Alanine, aspartate and glutamate metabolism	L-Glutamine ↓
20_P11 vs 20_P31	Phenylalanine metabolism Phenylalanine, tyrosine and tryptophan biosynthesis	Phenethylamine ↑ Shikimic acid ↓

P stress affected the accumulation of multiple flavonoid metabolites and the related processes of flavonoid metabolism in soybean roots [46]. In this study, we found that DMs participated in the flavonoid metabolism pathway in 10_P11 vs 10_P31, 20_P1 vs 20_P31 and 20_P11 vs 20_P31, but there were differences in type and quantity. Interestingly, we observed downregulated production of flavonoids fisetin and myricetin in the 10_P11 vs 10_P31 comparison, indicating altered flavonoid metabolic pathway activities. Similarly, downregulated production of naringenin, eriodictyol, and pinocembrin in 20_P1 vs 20_P31 and downregulated production of pinocembrin in 20_P11 vs 20_P31 indicated altered flavonoid metabolism pathway activities in roots exposed to prolonged P stress. Importantly, all of the abovementioned flavonoids have shown to exert antioxidant effects.

The observed downregulation of dopamine production in the P1 stress for 10 or 20 days suggests the involvement of the isoquinoline alkaloid biosynthesis-related pathway in the soybean root response to P stress. More studies have shown that dopamine can enhance tolerance to drought, salt stress, and nutrient deficiency in plants [61–63]. Dopamine possesses a strong antioxidative capacity comparable to glutathione (GSH) and certain flavonoids such as catechin and quercetin [64], and alleviates oxidative stress caused by different abiotic stressors by strengthening the antioxidant defense [65]. Therefore, the results of this study highlight a potential role for dopamine and the alkaloid biosynthetic pathway in the soybean root response to P stress.

It is noteworthy that β-nicotinamide mononucleotide was downregulated and participated in nicotinate and nicotinamide metabolism to adapt to the effects of P stress (P1 and P11) when subjected for 10 days. β-nicotinamide mononucleotide is a precursor required for NAD and NADP synthesis, which serve as key players in plant adaptations to stress by acting as coenzymes of various enzymes involved in numerous cellular metabolic pathways. Also regulating a wide range of cellular biochemical processes, including more than 300 redox reactions, where they play pivotal roles in maintaining intracellular redox states [66]. In fact, changes in NAD/NADP levels can alter intracellular redox states and cellular signal transduction pathways [67]. Additionally, NAD+ acts as an electron acceptor/donor in various energy-generating metabolic pathways, such as glycolysis, the TCA cycle, oxidative phosphorylation, and β-oxidation, which support numerous vitally important cellular biological processes [68]. In this study, β-nicotinamide mononucleotide was downregulated and participated in nicotinate and nicotinamide metabolism. Importantly, the downregulation of this DM subsequently led to reduced NAD/NADP production, resulting in altered expression of sugar-related metabolic pathways in soybean roots. Moreover, D-ribose and 6-phosphogluconic acid were downregulated and involved in the pentose phosphate pathway, in turn, influenced various cellular processes related to energy metabolism, growth, apoptosis, and others.

The TCA cycle is a central metabolic hub of respiration, nitrogen assimilation, and photorespiration pathways that provides energy for carbon metabolism and nitrogen metabolism [69]. Cis-aconitic acid is an intermediate involved in the conversion of citric acid to isocitric acid in the TCA cycle [70]. A previous study conducted by Rasouli et al. demonstrated that in high zinc-accumulating type of barley plants, enhanced zinc absorption by roots correlated with increased root secretion of malic acid, fumaric acid, and cis-aconitic acid [71]. Here we observed upregulated levels of cis-aconitic acid in P1 soybean roots exposed to P stress for 20 days. Due to cis-aconitic acid is a key participant in both the TCA cycle and glyoxylate and dicarboxylate metabolic pathways, these findings suggest that metabolic pathways associate with energy metabolism activate in soybean root exudates expose to P stress conditions, leading to increase energy production and secretion of substances that aid P-stressed plants in adapting to these disadvantage conditions. These results are the same as Tawaraya’s elucidated that acceleration of carbon flow to the TCA cycle was also performed in P-deficient soybean root [49].

Recent studies have highlighted the crucial roles of phospholipid-mediated signal transduction pathways in plant responses to various biotic and abiotic stresses [72–74]. These pathways also played a regulatory role in response to environmental changes, material absorption and secretion, energy conversion, and signal transduction [75, 76]. For example, phospholipid membrane components, which are vitally important for cellular activities of most organisms, play pivotal roles in interactions between rhizobia and leguminous plants, as well as in phospholipid signal transduction pathway-dependent activation of nodulation signal transduction pathways [77]. Mo found that under P conditions, the content of 23 metabolites related to lipid glycerophospholipids were significantly reduced in soybean roots, suggesting that P stress significantly promotes the degradation process of phospholipids [46]. The results obtained in this study indicate that P stress (P1 and P11) for 20 days, the phospholipid metabolism pathway was involved in the response of soybean roots to P stress. O-Phosphorylethanolamine was downregulated and participated in glycerophospholipid metabolism and sphingolipid metabolism to adapt or regulate the P stress.

## Conclusions

Low P stress significantly affected the types and quantities of root exudates in soybean, P1 treatment were mainly upregulated and P11 treatment were mainly downregulated. The metabolic pathways involved mainly included amino acid, flavonoid, and nicotinate and nicotinamide metabolic pathways and pathways related to isoquinoline alkaloid biosynthesis, sugar catabolism, and phospholipid metabolism, analyzed the physiological mechanisms of soybean root response to low P stress from the perspective of environmental adaptation, energy metabolism, and scavenging of reactive oxygen species through root exudates. These findings lay a foundation for further analyzing the impact of low P stress on the recognition of rhizobia, as well as the soybean nodule nitrogen fixation.

## Supporting information

S1 Fig Matchstick analysis of DMs. (DOCX)

S2 Fig Heatmap of correlation analysis of DMs. (DOCX)

S3 Fig Chord plot analysis of DMs. (DOCX)

S4 Fig KEGG classification of DMs. (DOCX)

S1 Table Details of metabolites. (XLSX)

S2 Table Details of DMs. (XLSX)

S3 Table Class and numbers of DMs. (DOCX)

S4 Table KEGG pathway and related compounds. (XLSX)

S5 Table Pathway and related compounds. (XLSX)
