# Supplementary material for: Response of soybean root exudates and related metabolic pathways to low phosphorus stress
Source: PLoS One. 2024 Dec 5;19(12):e0314256. doi: 10.1371/journal.pone.0314256 (PMC11620397; doi:10.1371/journal.pone.0314256)
Supplement: S1 Fig — (DOCX) [file pone.0314256.s001.docx]

10_P1 vs 10_P31


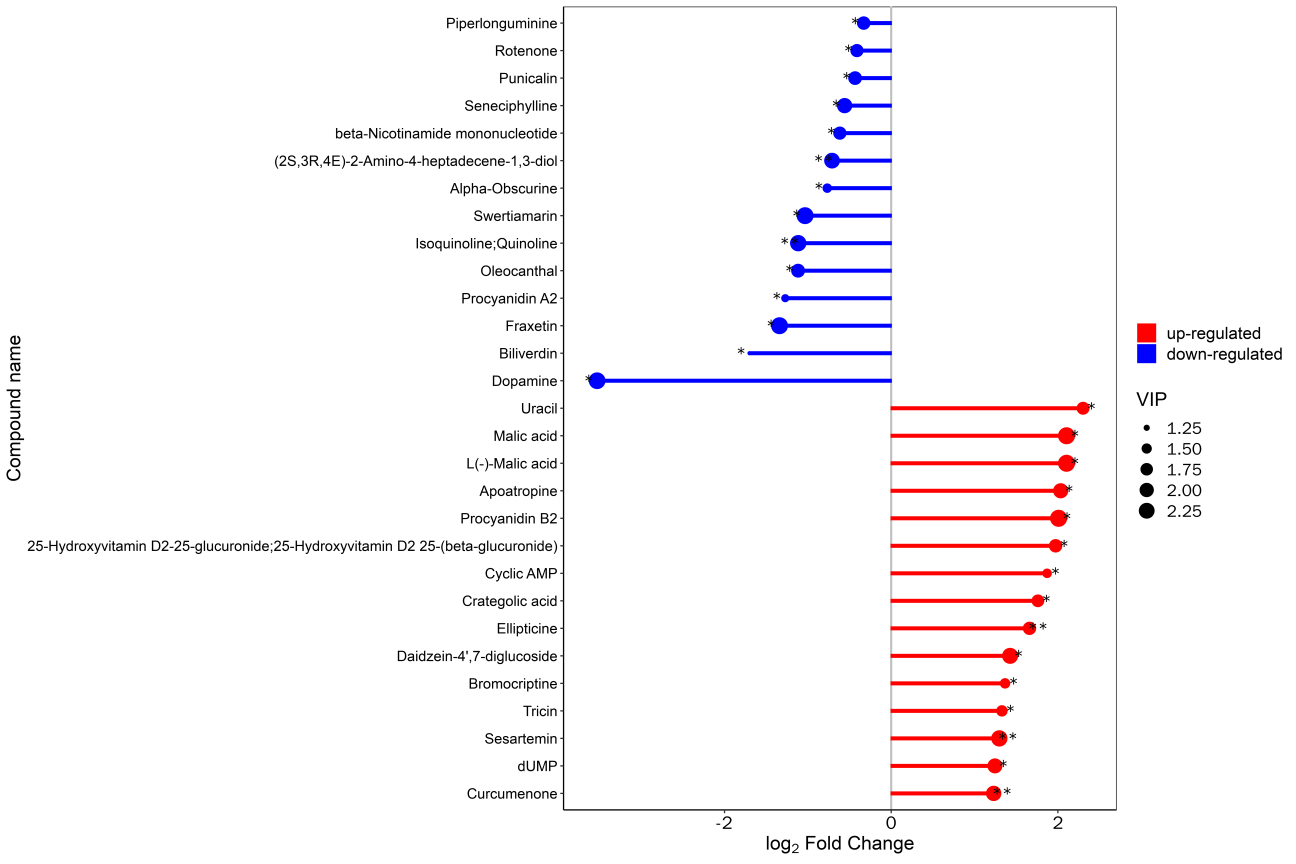


10_P11 vs 10_P31


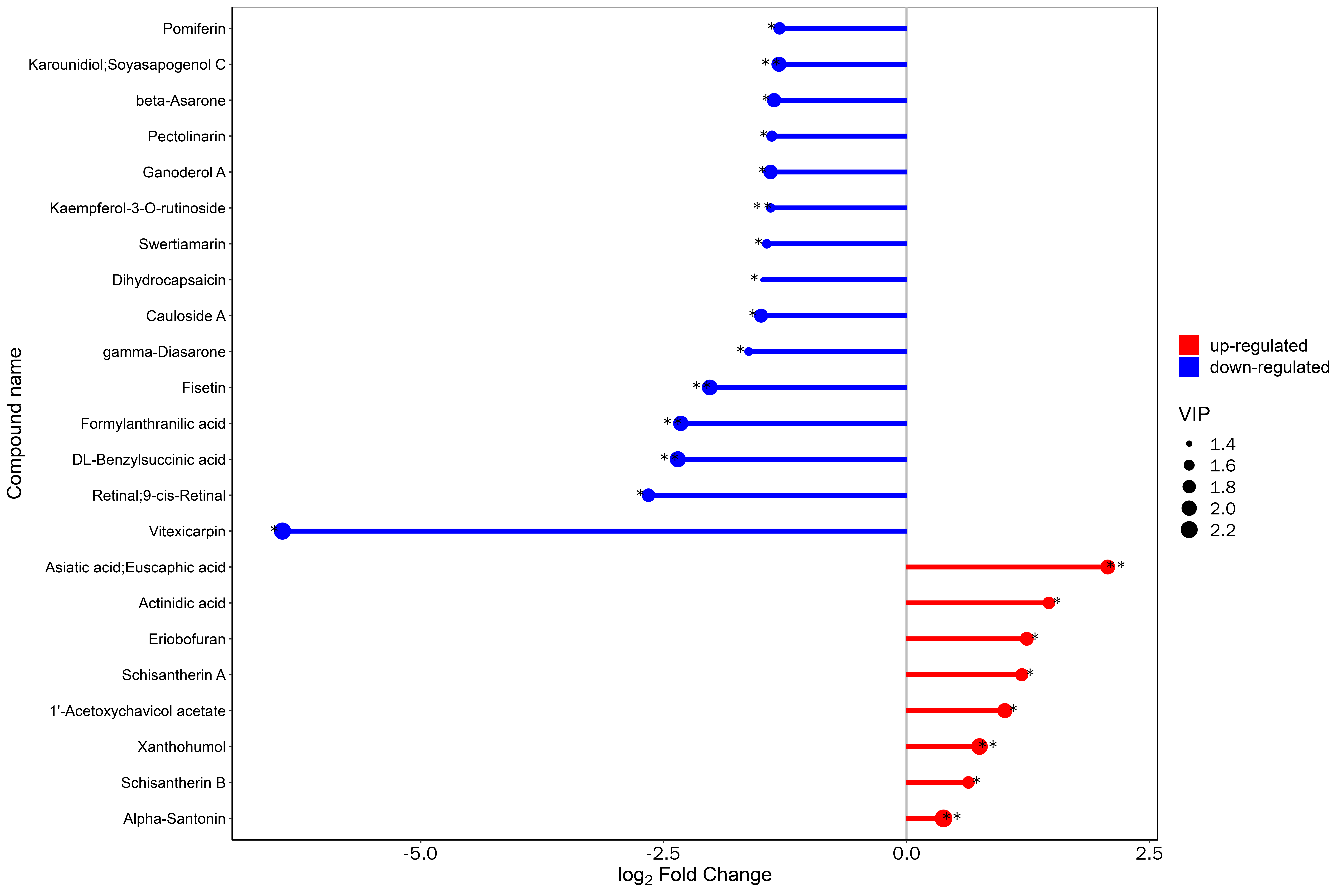


20_P1 vs 20_P31


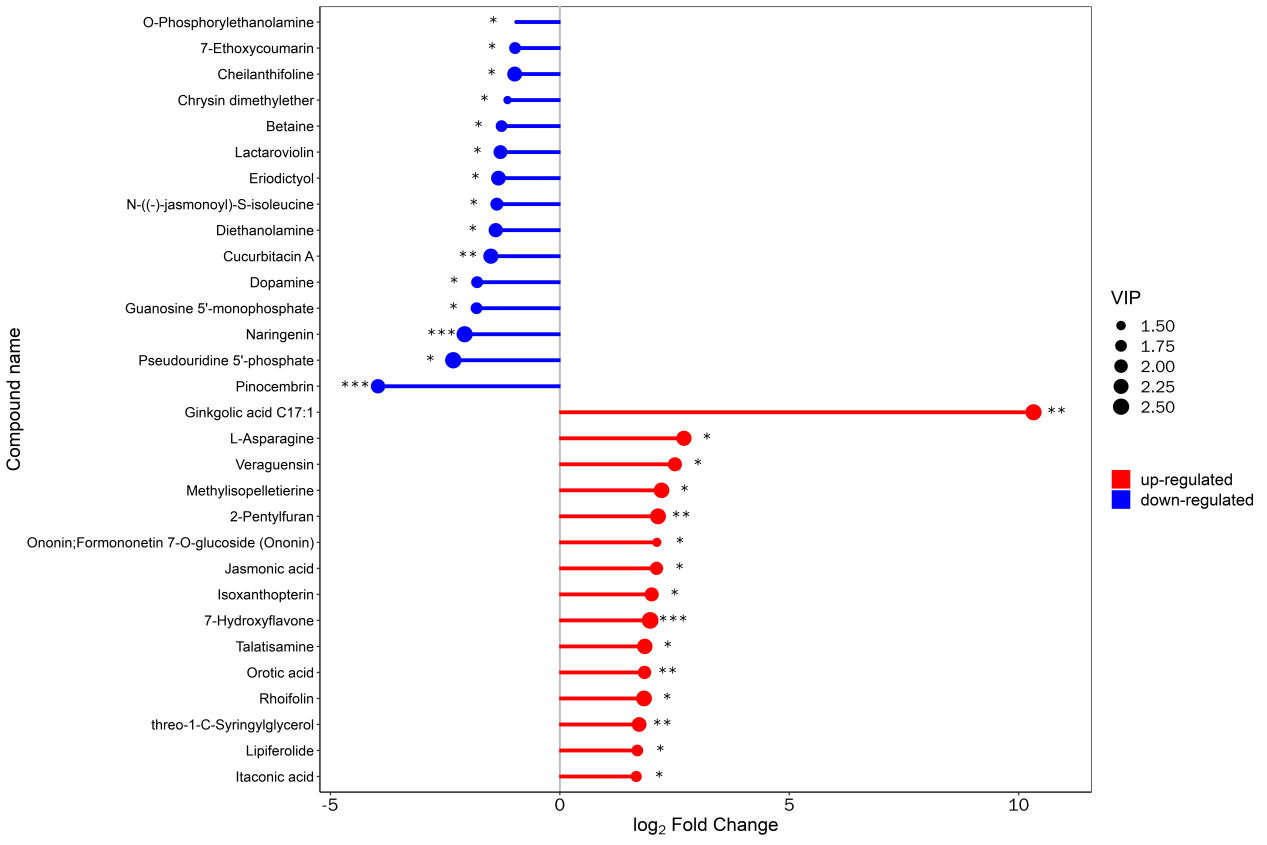


20_P11 vs 20_P31


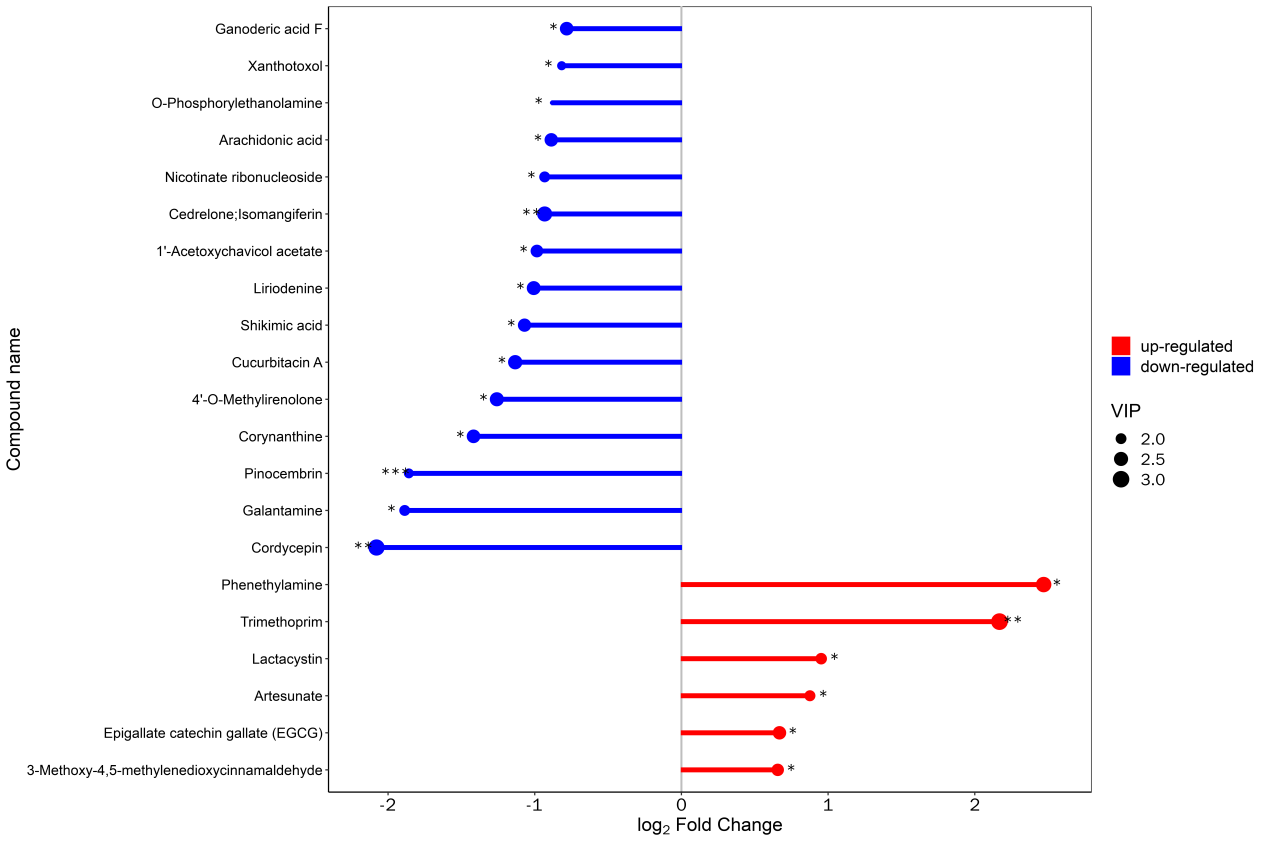


The abscissa shows the change multiple after logarithmic transformation, the shade of the dot represents the VIP value, and * represents the significance.

(Note: * 0.01<p<0.05, ** 0.001<p<0.01, *** p<0.001)
