# Supplementary material for: Response of soybean root exudates and related metabolic pathways to low phosphorus stress
Source: PLoS One. 2024 Dec 5;19(12):e0314256. doi: 10.1371/journal.pone.0314256 (PMC11620397; doi:10.1371/journal.pone.0314256)
Supplement: S2 Fig — (DOCX) [file pone.0314256.s002.docx]

10_P1 vs 10_P31


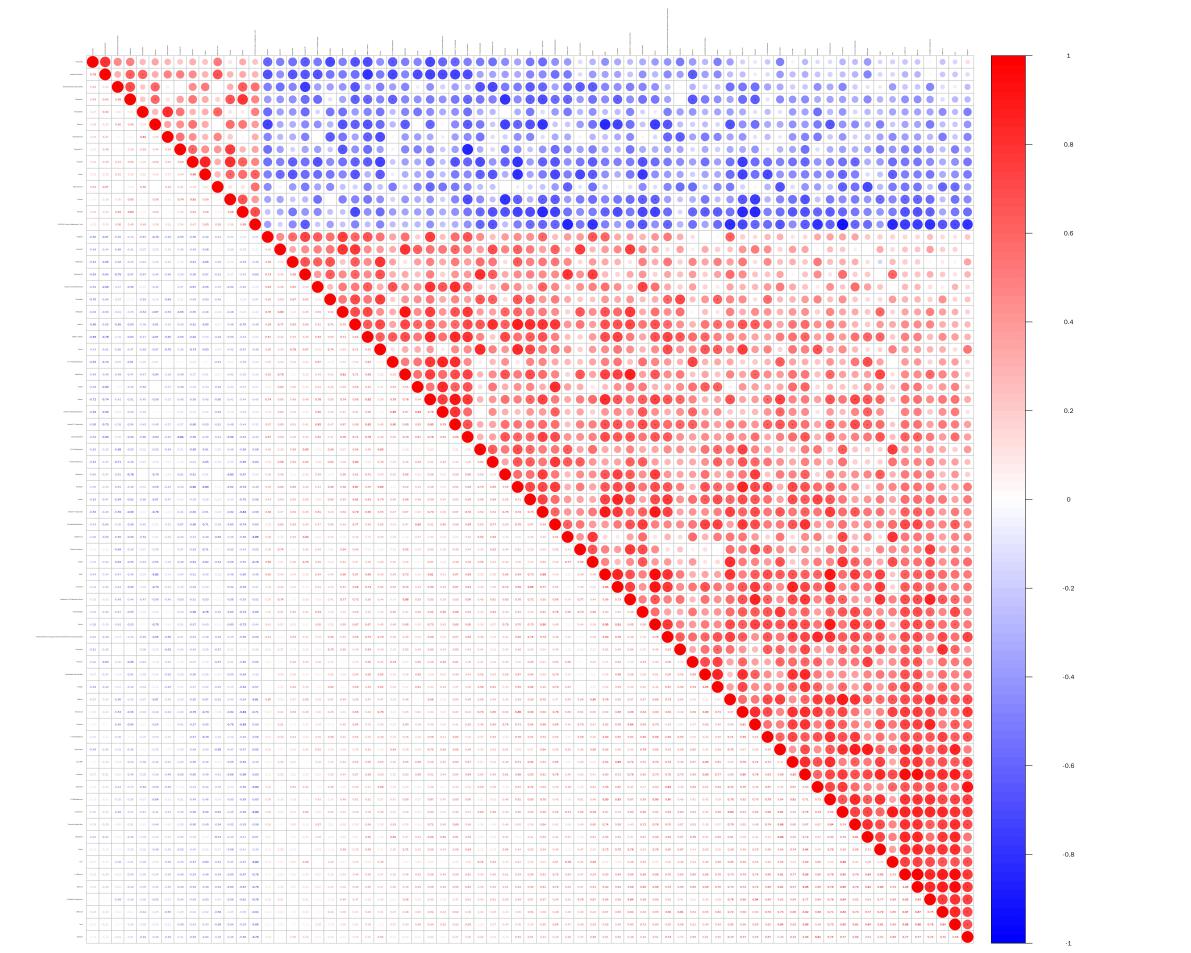


10_P11 vs 10_P31


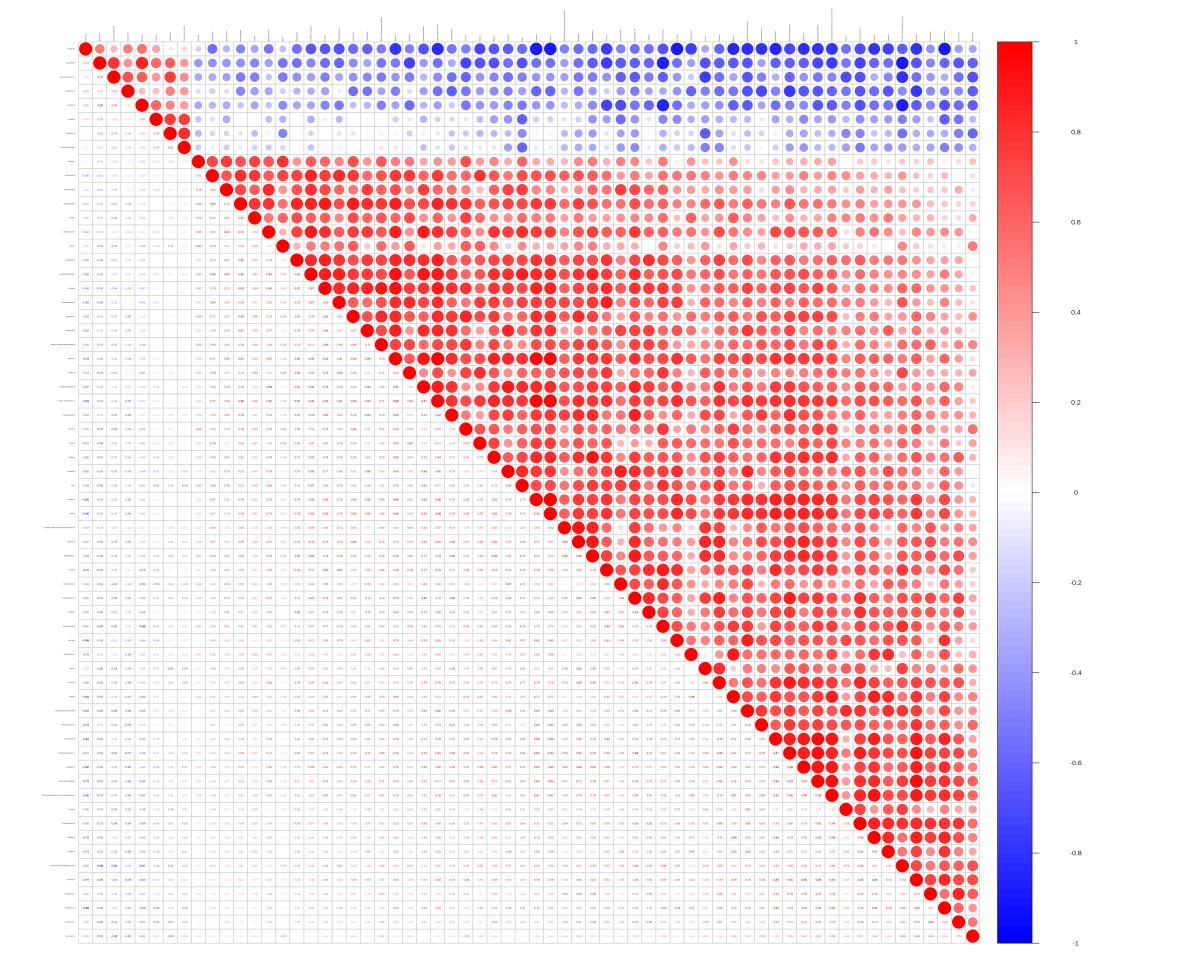


20_P1 vs 20_P31


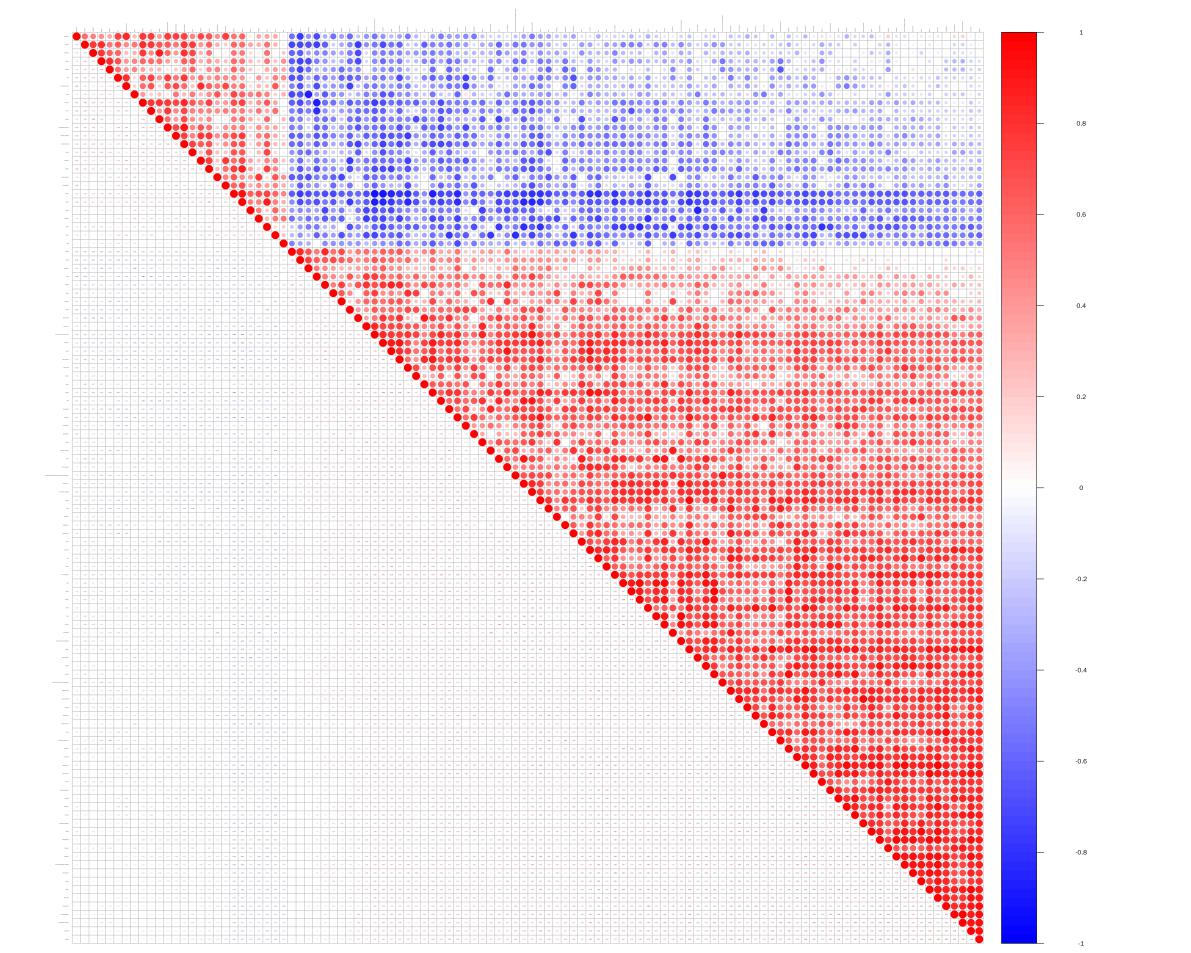


20_P11 vs 20_P31


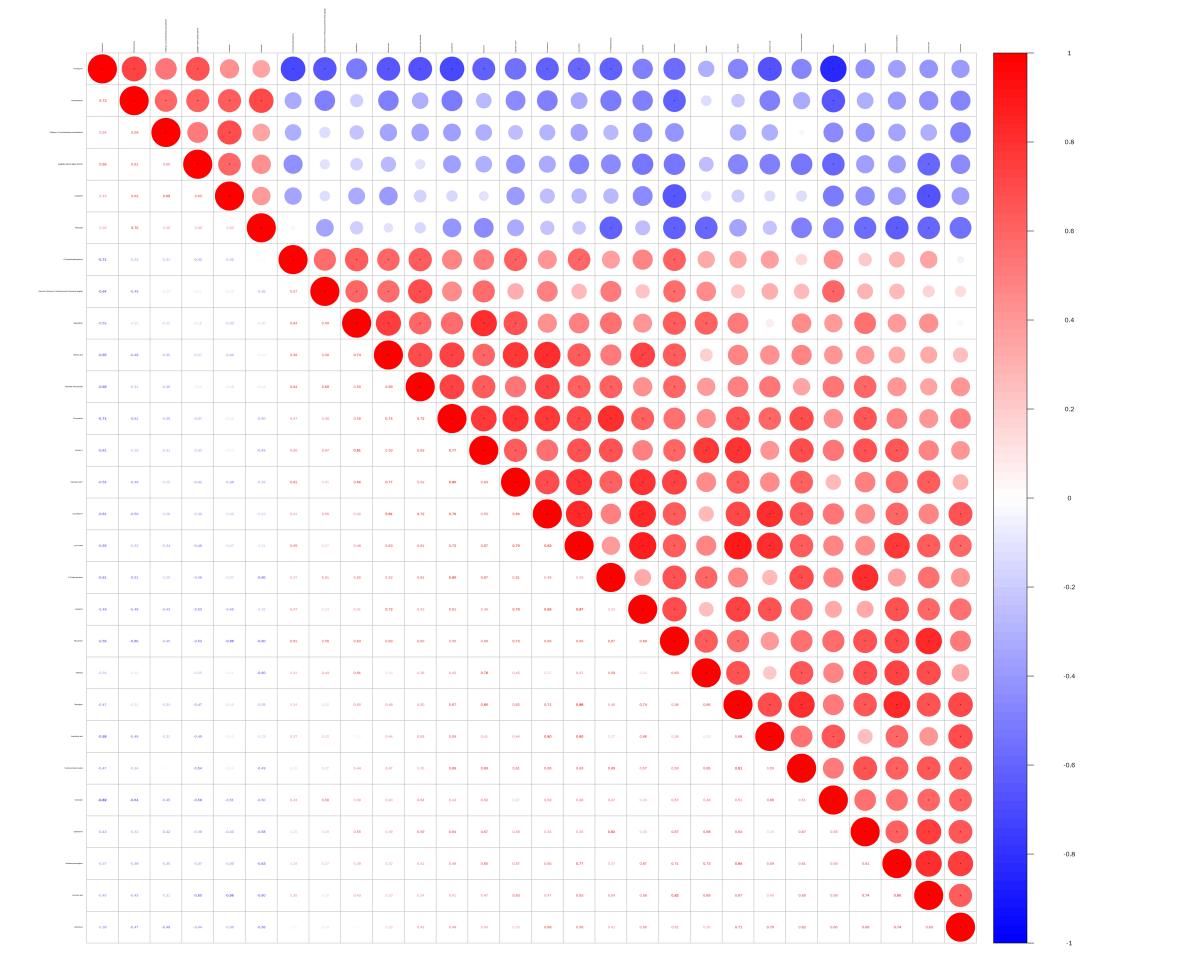


The horizontal and vertical coordinates represent the different metabolites of the comparison. The color patches at different locations represent the correlation coefficients between metabolites at the corresponding locations. Red represents positive correlation, blue represents negative correlation, and the darker the color, the stronger the correlation. * represents the significance.
