# Supplementary material for: Response of soybean root exudates and related metabolic pathways to low phosphorus stress
Source: PLoS One. 2024 Dec 5;19(12):e0314256. doi: 10.1371/journal.pone.0314256 (PMC11620397; doi:10.1371/journal.pone.0314256)
Supplement: S3 Fig — (DOCX) [file pone.0314256.s003.docx]

10_P1 vs 10_P31


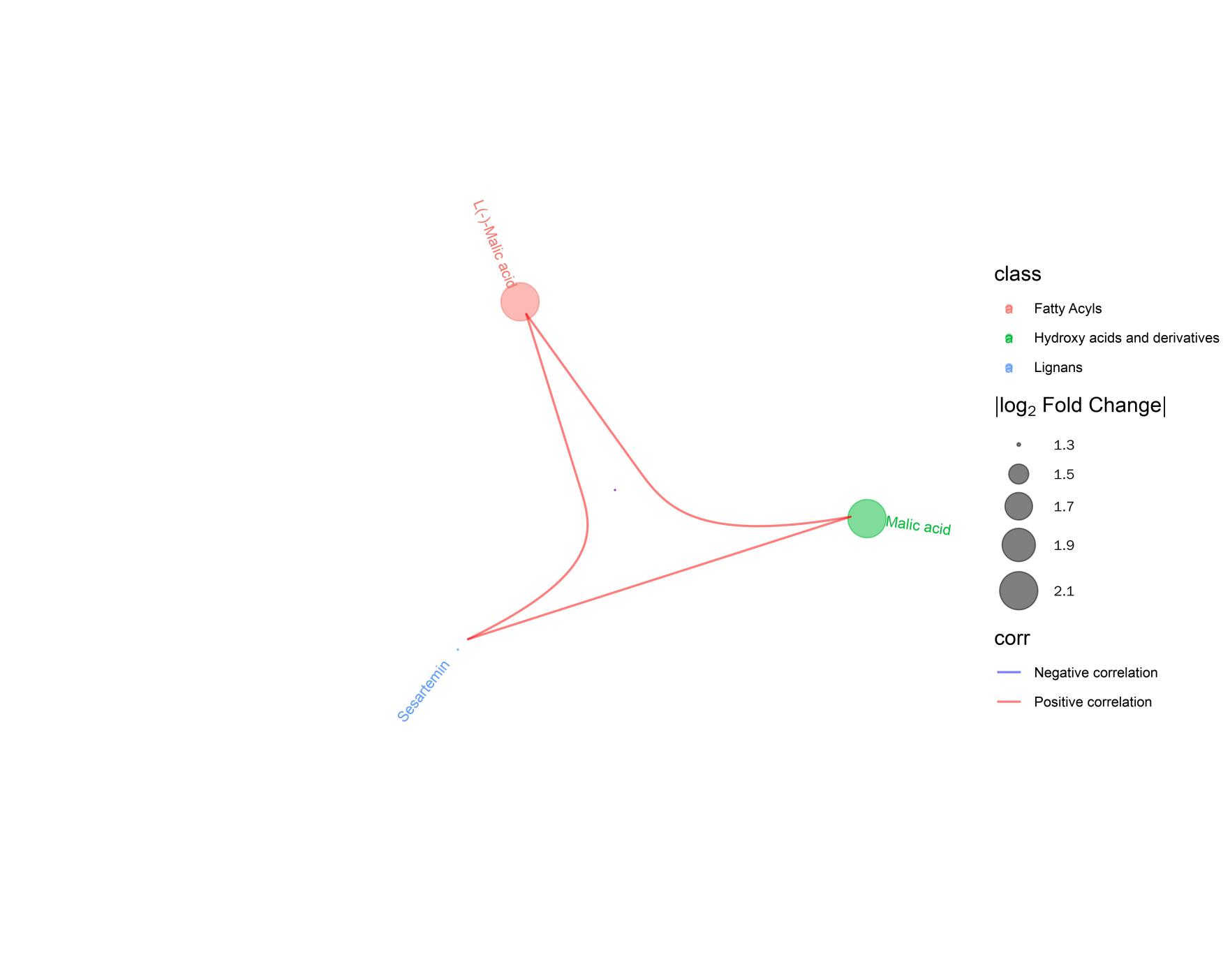


10_P11 vs 10_P31


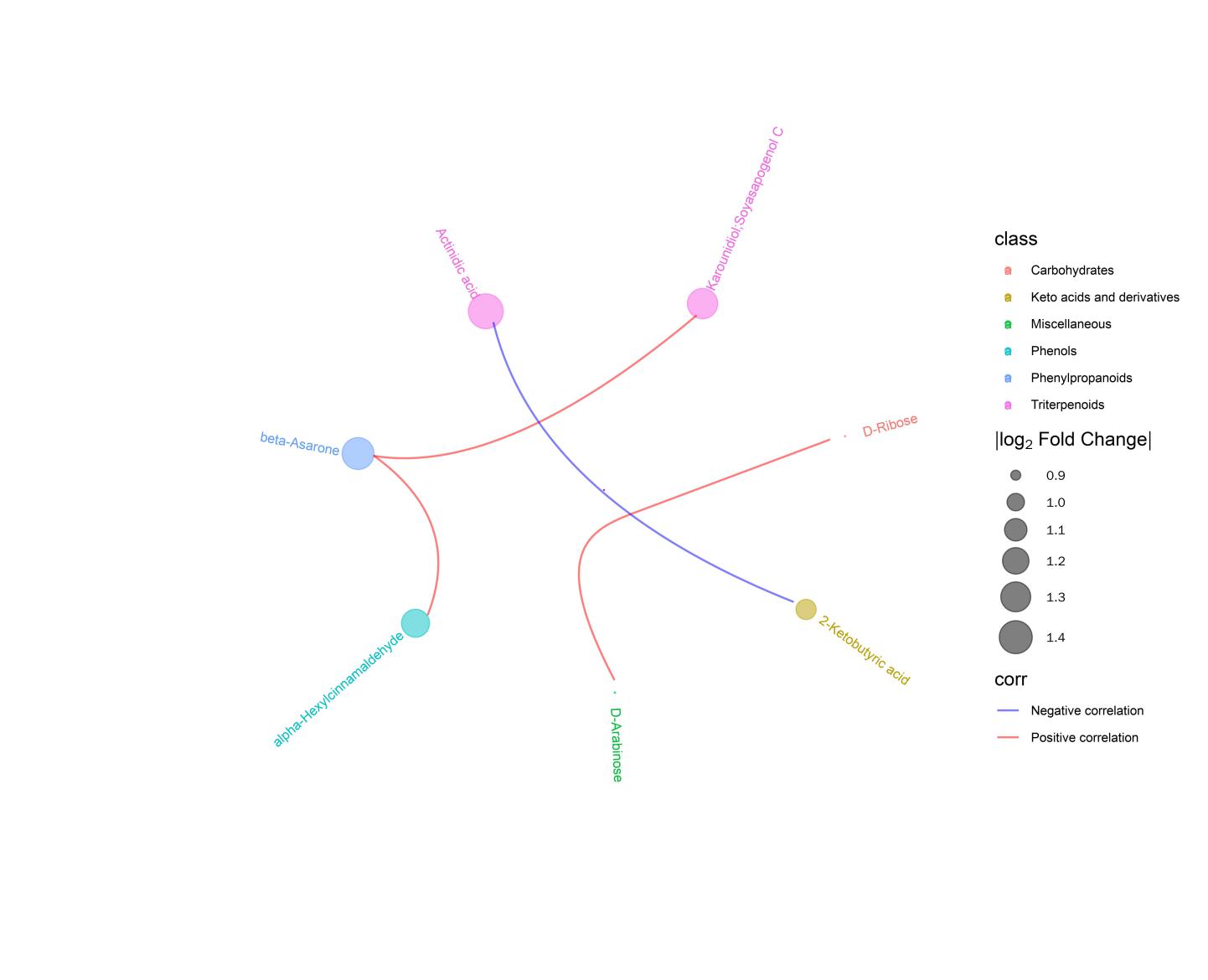


20_P1 vs 20_P31


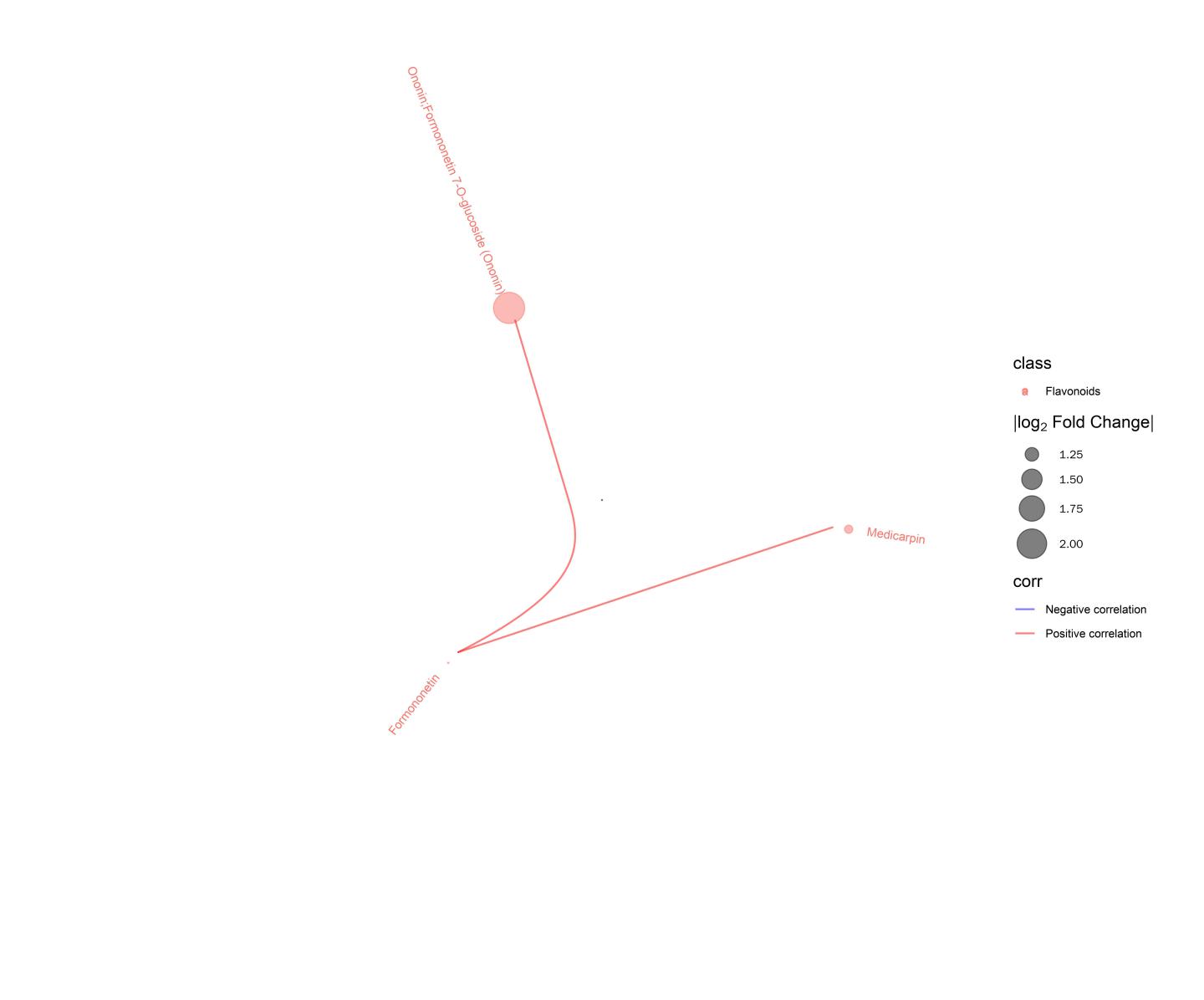


20_P11 vs 20_P31


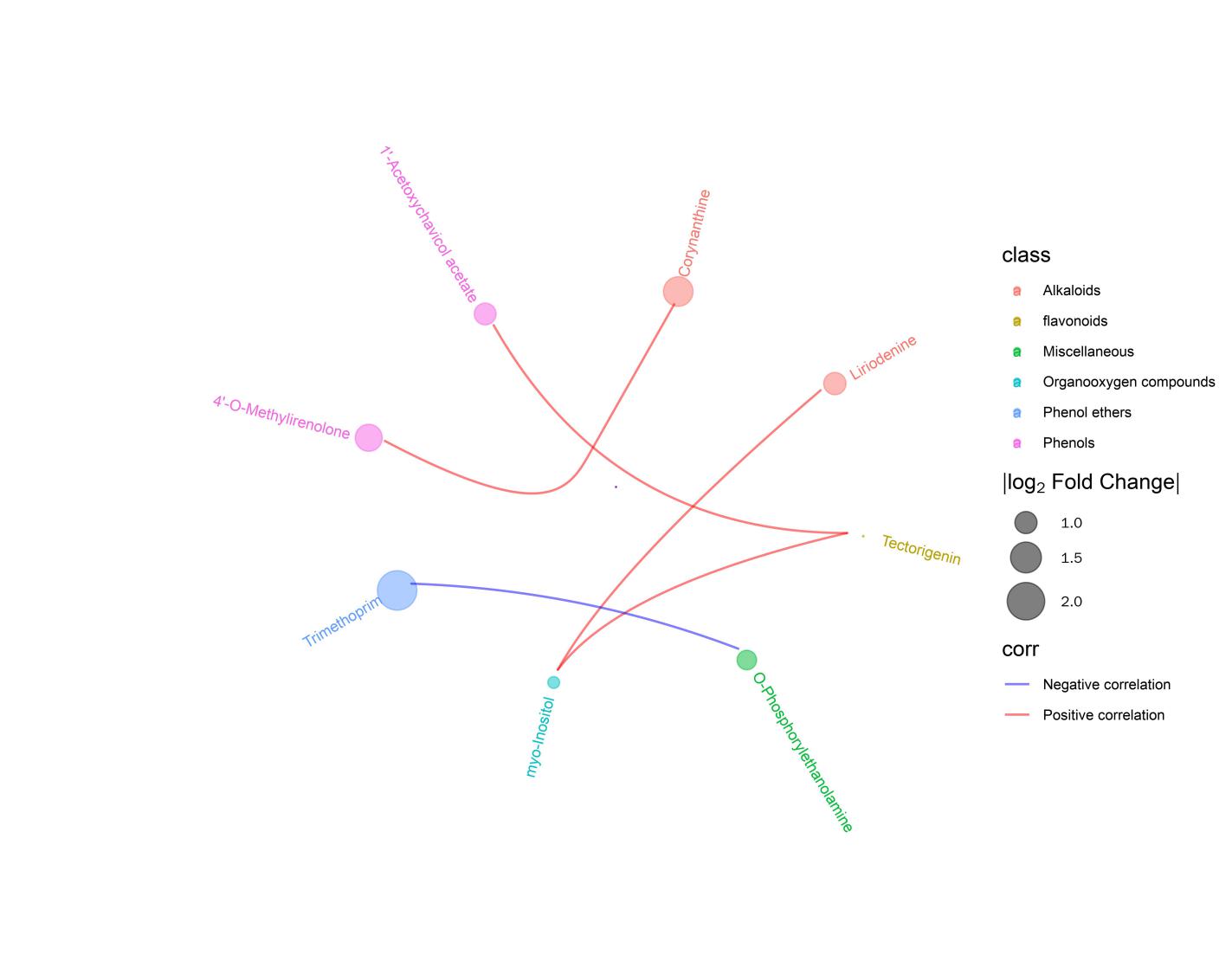


Point size represents the absolute value size of LOG-foldchange, the larger the point, the corresponding the absolute value of LOG-foldchange also increases. The color of the dot represents the source classification of the differential metabolites compared to the group, and the line represents the correlation coefficient value of the corresponding position metabolites.
