# Supplementary material for: Response of soybean root exudates and related metabolic pathways to low phosphorus stress
Source: PLoS One. 2024 Dec 5;19(12):e0314256. doi: 10.1371/journal.pone.0314256 (PMC11620397; doi:10.1371/journal.pone.0314256)
Supplement: S4 Fig — (DOCX) [file pone.0314256.s004.docx]

10_P1 vs 10_P31


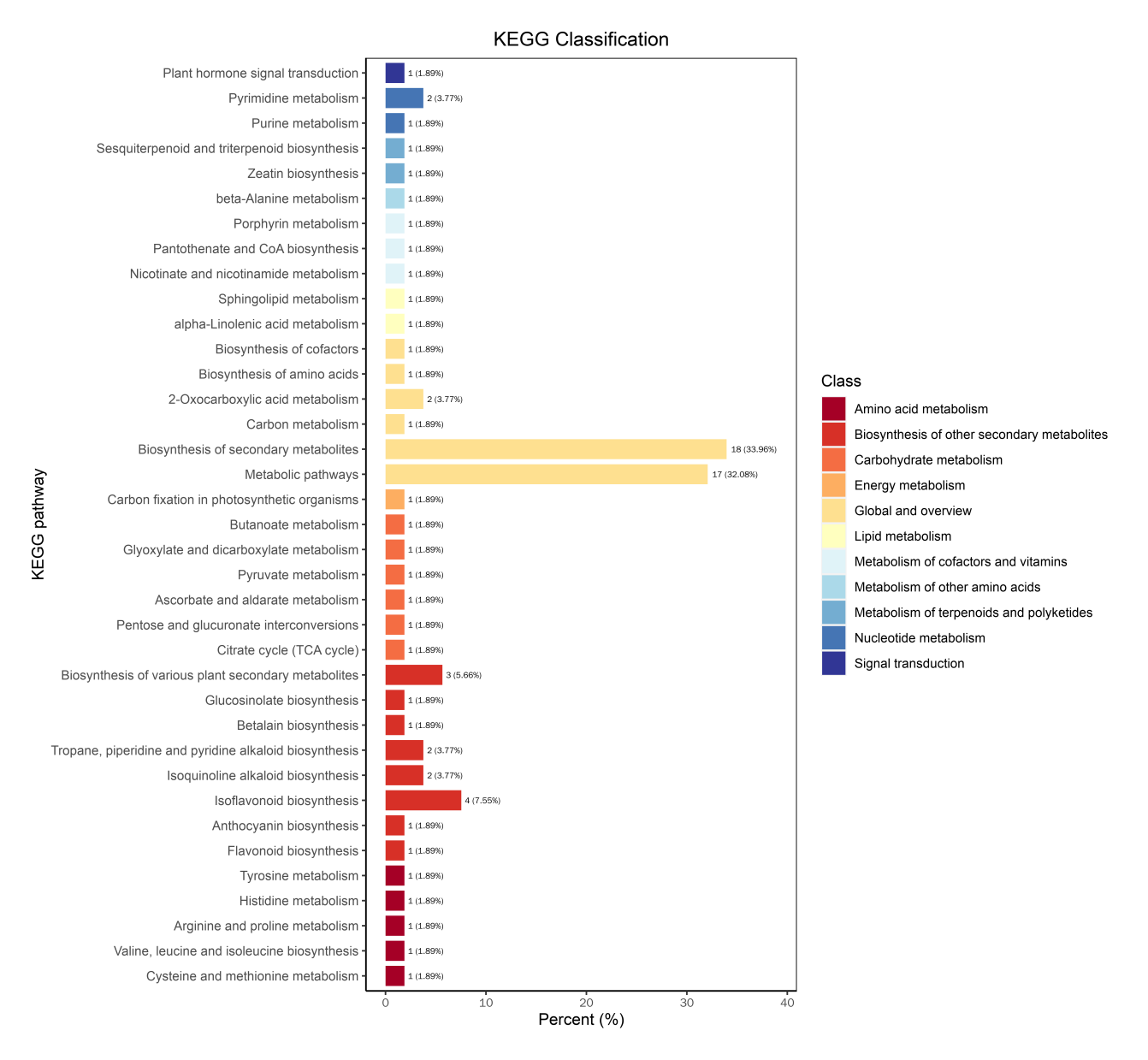


10_P11 vs 10_P31


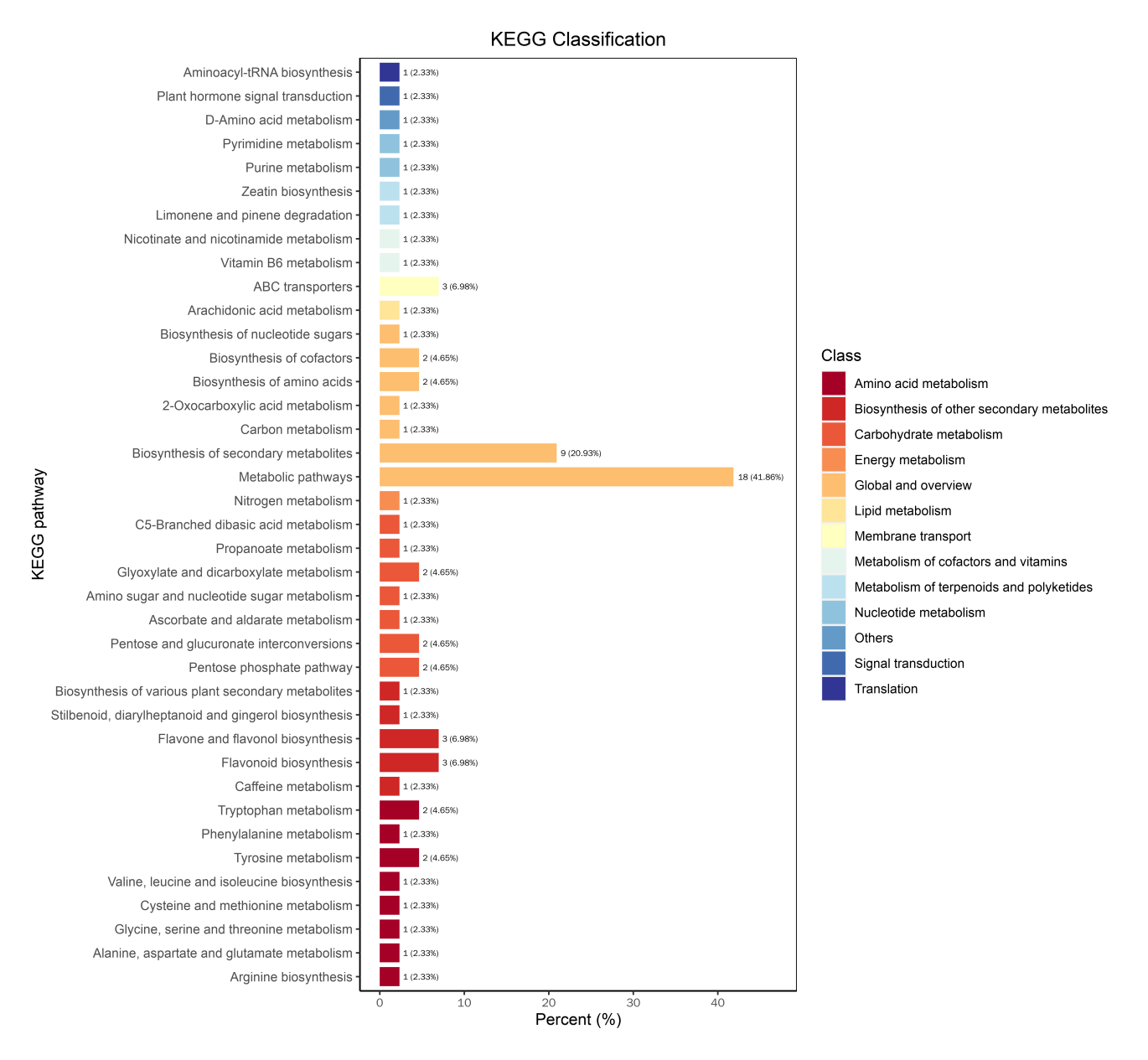


20_P1 vs 20_P31


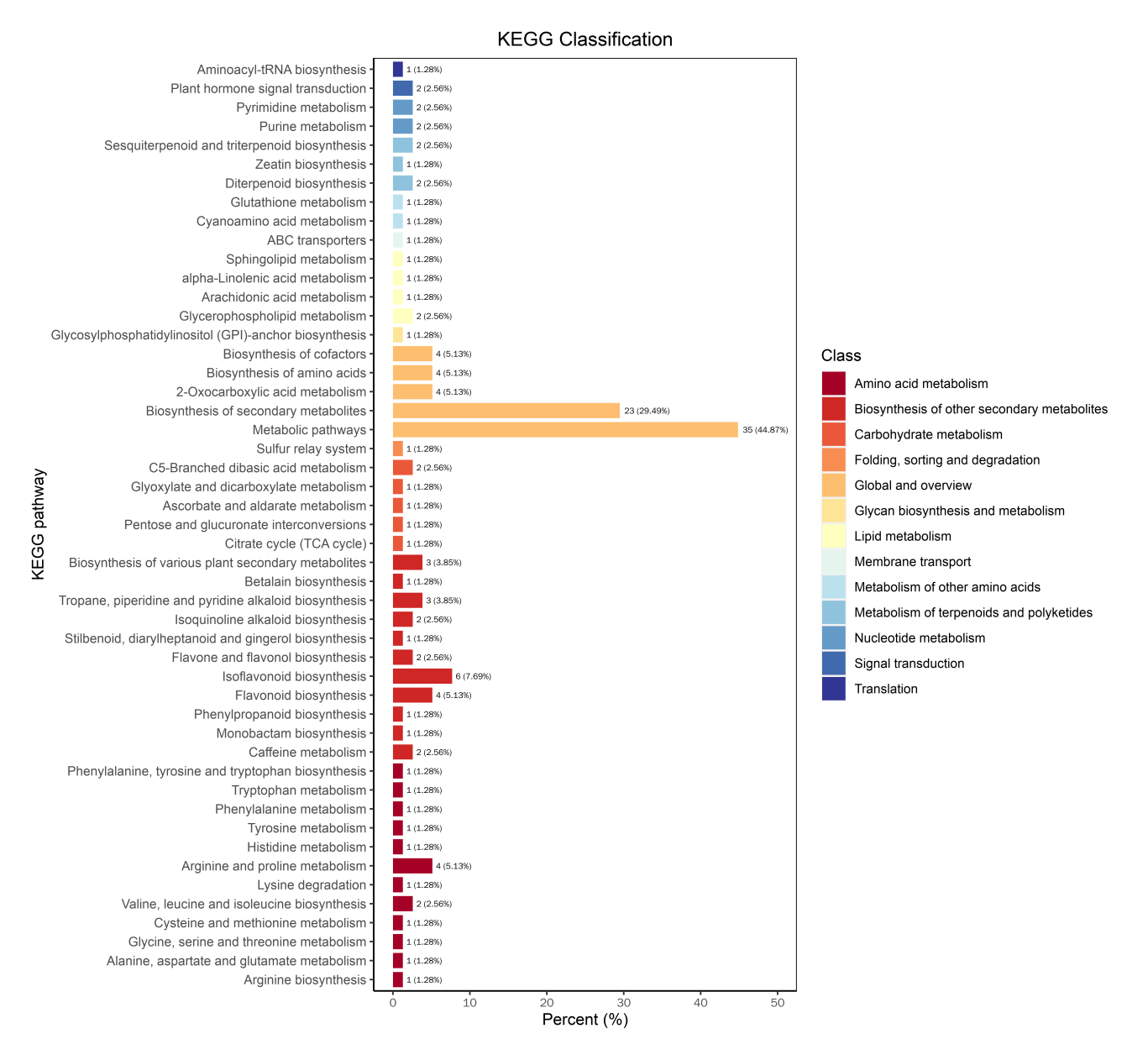


20_P11 vs 20_P31


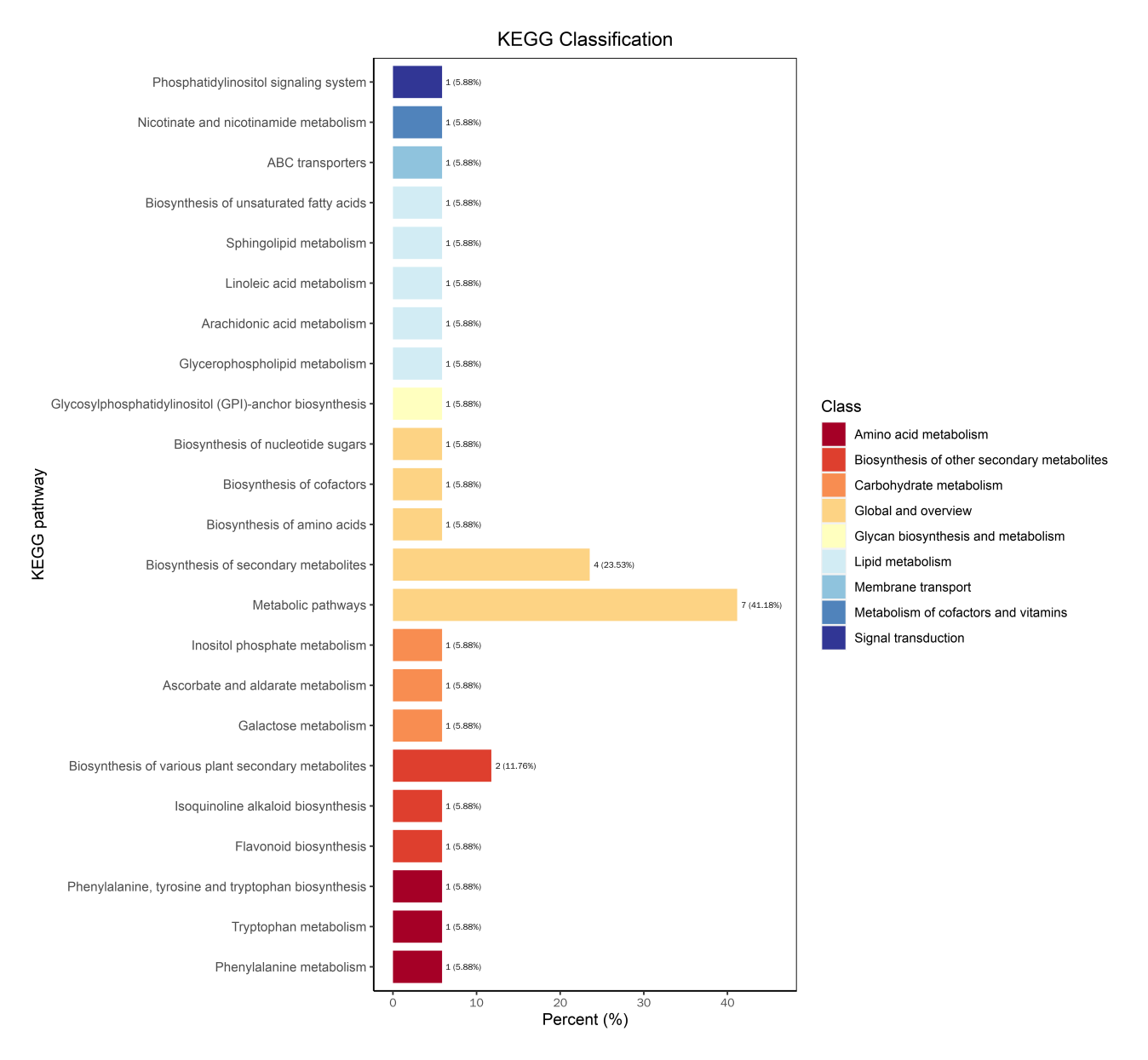


The horizontal axis represents the percentage of annotated metabolites under a certain pathway to all annotated metabolites, while the vertical axis represents the enriched KEGG metabolic pathway ID number.
