# Supplementary material for: Response of soybean root exudates and related metabolic pathways to low phosphorus stress
Source: PLoS One. 2024 Dec 5;19(12):e0314256. doi: 10.1371/journal.pone.0314256 (PMC11620397; doi:10.1371/journal.pone.0314256)
Supplement: S3 Table — (DOCX) [file pone.0314256.s007.docx]

Supplementary Table 3

|  |  | Class | numbers | up - regulated | down- regulated |  | Class | numbers | up - regulated | down- regulated |
| --- | --- | --- | --- | --- | --- | --- | --- | --- | --- | --- |
| 10_P1 vs 10_P31 | 1 | Flavonoids | 6 | 5 | 1 | 9 | Pyrimidine nucleotides | 1 | 1 | 0 |
|  | 2 | Alkaloids | 4 | 3 | 1 | 10 | Hydroxy acids and derivatives | 1 | 1 | 0 |
|  | 3 | Terpenoids | 3 | 3 | 0 | 11 | Tetrapyrroles and derivatives | 1 | 0 | 1 |
|  | 4 | Phenols | 2 | 0 | 2 | 12 | Coumarins | 1 | 0 | 1 |
|  | 5 | Nucleotide and its derivates | 2 | 2 | 0 | 13 | Organooxygen compounds | 1 | 1 | 0 |
|  | 6 | Lignans | 2 | 2 | 0 | 14 | Steroids | 1 | 1 | 0 |
|  | 7 | Iridoids | 1 | 0 | 1 | 15 | Fatty Acyls | 1 | 1 | 0 |
|  | 8 | Ergoline and derivatives | 1 | 1 | 0 |  |  |  |  |  |
| 10_P11 vs 10_P31 | 1 | Terpenoids | 8 | 2 | 6 | 10 | Nucleotide and its derivates | 1 | 0 | 1 |
|  | 2 | Flavonoids | 6 | 0 | 6 | 11 | Iridoids | 1 | 0 | 1 |
|  | 3 | Phenols | 5 | 2 | 3 | 12 | Tryptamine derivatives | 1 | 0 | 1 |
|  | 4 | Phenylpropanoids | 3 | 0 | 3 | 13 | Alkaloids | 1 | 0 | 1 |
|  | 5 | Lignans | 3 | 1 | 2 | 14 | Keto acids and derivatives | 1 | 0 | 1 |
|  | 6 | Organooxygen compounds | 2 | 0 | 2 | 15 | Prenol lipids | 1 | 0 | 1 |
|  | 7 | Steroids and steroid derivatives | 2 | 0 | 2 | 16 | Fatty Acyls | 1 | 0 | 1 |
|  | 8 | Amino acid and derivatives | 1 | 0 | 1 | 17 | Phytohormone | 1 | 0 | 1 |
|  | 9 | Benzene and substituted derivatives | 1 | 0 | 1 | 18 | Benzothiazoles | 1 | 0 | 1 |
| 20_P1 vs 20_P31 | 1 | Terpenoids | 9 | 8 | 1 | 10 | Pteridines and derivatives | 1 | 1 | 0 |
|  | 2 | Alkaloids | 8 | 6 | 2 | 11 | Nucleotide and its derivates | 1 | 0 | 1 |
|  | 3 | Flavonoids | 12 | 8 | 4 | 12 | Quinolines and derivatives | 1 | 1 | 0 |
|  | 4 | Phenols | 4 | 3 | 1 | 13 | Lignans | 1 | 1 | 0 |
|  | 5 | Organic acids and derivatives | 3 | 3 | 0 | 14 | Miscellaneous | 2 | 2 | 0 |
|  | 6 | Fatty Acyls | 3 | 3 | 0 | 15 | Tryptamine derivatives | 1 | 1 | 0 |
|  | 7 | Phenylpropanoids | 2 | 2 | 0 | 16 | Vitamins | 1 | 1 | 0 |
|  | 8 | Prenol lipids | 2 | 1 | 1 | 17 | Organooxygen compounds | 1 | 0 | 1 |
|  | 9 | Amino acid and derivatives | 1 | 1 | 0 | 18 | phytohormone | 1 | 0 | 1 |
| 20_P11 vs 20_P31 | 1 | Alkaloids | 3 | 0 | 3 | 5 | Flavonoids | 1 | 0 | 1 |
|  | 2 | Phenols | 2 | 1 | 1 | 6 | Terpenoids | 1 | 0 | 1 |
|  | 3 | Benzene and substituted derivatives | 1 | 1 | 0 | 7 | Organic acids and derivatives | 1 | 0 | 1 |
|  | 4 | Nucleotide and its derivates | 1 | 0 | 1 |  |  |  |  |  |
